# Liquid Chromatographic Quadrupole Time-of-Flight Mass Spectrometric Untargeted Profiling of (Poly)phenolic Compounds in *Rubus idaeus* L. and *Rubus occidentalis* L. Fruits and Their Comparative Evaluation

**DOI:** 10.3390/antiox10050704

**Published:** 2021-04-29

**Authors:** Lapo Renai, Cristina Vanessa Agata Scordo, Ugo Chiuminatto, Marynka Ulaszewska, Edgardo Giordani, William Antonio Petrucci, Francesca Tozzi, Stefania Nin, Massimo Del Bubba

**Affiliations:** 1 Department of Chemistry “Ugo Schiff”, University of Florence, 50019 Sesto Fiorentino, Italy; lapo.renai@unifi.it (L.R.); cscordo@unifi.it (C.V.A.S.); 2 Sciex Europe, Landwehrstraße 54, 64293 Darmstadt, Germany; ugo.chiuminatto@sciex.com; 3 Center for Omics Sciences, Proteomics and Metabolomics Facility-ProMeFa, IRCCS San Raffaele Scientific Institute, 20132 Milan, Italy; ulaszewska.maria@hsr.it; 4 Department of Agriculture, Food, Environment and Forestry, 50019 Sesto Fiorentino, Italy; edgardo.giordani@unifi.it; 5 Research Centre for Viticulture and Enology, Council for Agricultural Research and Economics, 2100 Arezzo, Italy; williamantonio.petrucci@crea.gov.it; 6 Research Centre for Vegetable and Ornamental Crops, Council for Agricultural Research and Economics, 51017 Pescia, Italy; francesca.tozzi@crea.gov.it (F.T.); stefania.nin@crea.gov.it (S.N.)

**Keywords:** raspberry, metabolomics, high resolution mass spectrometry, data independent acquisition, phenolic compounds, pomological characterization

## Abstract

This study provided a detailed profiling of the antioxidant and bioactive compounds occurring in three varieties of *Rubus idaeus* L. fruits (“Fall Gold”, “Glen Ample” and “Tulameen”) compared to *Rubus occidentalis* L. black raspberry (“Jewel” cultivar), adopting a comprehensive untargeted metabolomics approach developed with UHPLC analysis coupled with quadrupole/time-of-flight high resolution mass spectrometry, using the SWATH^®^ acquisition protocol. The feature selection and annotation workflow, applied to the analysis of raspberry extracts in both polarities, allowed identifying 68 bioactive compounds mainly belonging to the classes of (poly)phenolic compounds. Interestingly, some of these identifications (e.g., ferulic acid glycosides and the ellagitannin-like nobotanin/malabathrin) represent the first report in raspberry fruits. Principal component analysis made possible highlighting the features more related to the expression of a genotype effect within the *R. idaeus* species or between the two raspberry species herein investigated. Overall, flavanols were the most discriminating features for the Fall Gold variety, whereas ellagitannins and flavonol glycosides represent more distinctive metabolic traits in Glen Ample and Tulameen fruits. Moreover, *R. occidentalis* Jewel variety was strongly characterized by the occurrence of anthocyanins, such as cyanidin, pelargonidin and delphinidin glycosides.

## 1. Introduction

Wild berries are well-known sources of bioactive compounds, mainly phenolics, synthesized by the plant secondary metabolism [1,2,3]. Such native chemicals play a crucial role in the prevention of a wide range of chronic and degenerative diseases [4,5]. For example, bilberry, blueberry and raspberry extracts demonstrated to exert antiproliferative and/or pro-apoptotic effects on specific cancer cell lines [6,7,8], whereas the intake of blueberry and strawberry supplements showed beneficial effects in the prevention and treatment of cardiovascular diseases [9] and diabetes [10], respectively.

Within the wide variety of wild berries, raspberry and blackberry are the most consumed edible fruits of a multitude of plant species in the genus *Rubus* of the *Rosaceae* family [11]. *Rubus* fruits are considered important functional foods due to their remarkable nutritional value and content of dietary antioxidants [11,12]. Within *Rubus* species, blackberry is commonly harvested as spontaneous wild berry, while raspberry is widely cultivated in Europe and North America as *Rubus idaeus* L. and *Rubus occidentalis* L. species, respectively [13], with a worldwide annual production of about one million tons in 2019 [14]. Despite their significant commercial value and recognized dietary importance, raspberry fruits have been investigated for their bioactive phenolic constituents to a limited extent.

More in detail, the phenolic composition of *R. occidentalis* berries has been poorly described in the literature, since, to our knowledge, only three studies have been published thus far [2,15,16], while a few more studies have been performed on *R. idaeus* fruits [2,17,18,19,20,21,22,23]. Some of these studies have been performed using liquid chromatography (LC) coupled with diode array detection and single quadrupole mass spectrometry through a selected ion monitoring approach, highlighting the occurrence of anthocyanins (i.e., cyanidin and pelargonidin derivatives) [2,19] and 32 compounds belonging to phenolic acids, flavanols, flavonols, ellagic acid and its derivatives and ellagitannins [2]. Other studies [17,18,20,21] adopted LC coupled with linear ion trap low-resolution mass spectrometer, which is in principle capable of more-in-depth structural analyses compared to single quadrupole. In this regard, a significant improvement of the identification process was obtained by Dincheva and co-workers [18], thus pushing the annotation up to 60 compounds belonging to the flavonols, flavanols and hydroxycinnamic acids, in addition to anthocyanins and ellagitannins. However, the use of low-resolution mass spectrometry does not provide the exact mass of both precursor and fragment ions, thus resulting in a low-accuracy identification, unless authentic standards are available. Conversely, high-resolution mass spectrometry, especially in the tandem mode (e.g., quadrupole/time-of-flight or quadrupole-orbital trap), is much more informative, since it allows obtaining important structurally related information through the fragmentation of parent molecules and the accurate mass readout of both precursors and fragments [1,24]. This consideration highlights the importance of adopting acquisition strategies based on high-resolution mass spectrometry, which has been applied in few cases for the exploration of the phenolic fraction of raspberry fruits, using LC hyphenated with time-of-flight [22] or quadrupole/time-of-flight (Q/TOF) [15,23] mass spectrometry (MS). More in detail, untargeted LC-Q/TOF was recently adopted for investigating negatively ionizable phenolic compounds in *R. occidentalis* berries [15]. Despite the accurate workflow adopted for feature extraction and annotation, the list of identified analytes was limited to a small number of compounds belonging to phenolic acids, flavonols, flavanols, ellagic acid and ellagic acid glycosides. A deeper untargeted analysis was performed by Zhang and co-workers [23] on *R. idaeus* extracts, identifying 50 phenolic secondary metabolites included in the group of anthocyanins and in the other aforementioned categories.

The expression of phenolic compounds in *R. idaeus* raspberries has been investigated as a function of stage of maturity [17], growing region [22] and genotype [17,22,25], suggesting the last one as the variable most influencing the phenolic profile. However, no data are reported in the literature concerning the effect of genotype between the *R. idaeus* and *R. occidentalis* berry species.

Based on the aforementioned considerations, this paper aims at: (i) deepening the knowledge of the phenolic fraction of *R. idaeus* and *R. occidentalis* berries, the latter being poorly described in the literature; and (ii) investigating the genotype effect on the phenolic expression in the two species. To these purposes, an untargeted metabolomics LC-Q/TOF study was performed on berries from “Glen Ample”, “Tulameen” and “Fall Gold” varieties of *R. idaeus* and from “Jewel” cultivar of *R. occidentalis*, cultivated in the same orchard and under the same rural practices.

## 2. Materials and Methods

### 2.1. Reagents and Standards

Analytical reference standards for identity confirmation were supplied, as specified in Section 1 of the Supplementary Materials. Hydrochloric acid (37%), LC-MS grade methanol and water were obtained from J.T. Baker (Deventer, the Netherlands). HPLC grade acetone, glacial acetic acid and LC-MS formic acid were purchased from Sigma-Aldrich (St. Louis, MO, USA). Sodium fluoride, Folin–Ciocalteu reagent and sodium carbonate were obtained by Merck (Darmstadt, Germany). Ultrapure water was taken from a Milli-Q system (Millipore, Billerica, MA, USA). Nylon membranes (porosity 0.2 μm) for the filtration of raspberry extracts before HPLC analysis were obtained from VWR™ International (Radnor, PA, USA).

### 2.2. Sample Preparation

*R. idaeus* and *R. occidentalis* analyzed in the present study were grown in a same experimental site located in Tuscany (44°02.175′, 10°47.361 ′, altitude 450 m a.s.l.), under the same agricultural conditions. The cultivars of *R. idaeus* investigated were “Glen Ample” (GA, red), “Tulameen” (T, red) and “Fall Gold” (G, yellow), whereas, for *R. occidentalis,* the cultivar “Jewel” (J, black) was selected. Six independent samples, each consisting of ten fresh fruits free of defects, were prepared for each raspberry cultivar. Three samples were used for the untargeted LC-Q/TOF study, while the others were destined to the pomological analyses. After the sampling, the berries intended for extraction were immediately frozen in liquid nitrogen, freeze-dried, grinded to obtain a homogeneous powder and finally stored at −20 °C until extraction and analysis were performed. Three representative aliquots from each freeze-dried berry sample were extracted according to a procedure previously developed by Zhang and co-workers [23] with few modifications. Briefly, about 500 mg dry weight (d.w.) raspberry aliquots were mixed with 5 mL of acetone/10 mM NaF water/acetic acid (70:29.7:0.3, *v*/*v*/*v*) and then vortexed for 30 s, sonicated in ice bath at controlled temperature (~0 °C) for 5 min and centrifuged at 8000× *g* for 10 min. This extraction protocol was replicated four times and its efficiency evaluated by measuring spectrophotometrically total soluble polyphenols (TSP) and total monomeric anthocyanins (TMA), as reported by Renai et al. [26] (see Section 2 of the Supplementary Materials for full details), using calibration curve ranges of 5–10 µg of procyanidin B1 and 20–300 µg of cyanidin-3-*O*-sophoroside, respectively. The results show that the fourth extraction allowed for recovering 3.6–6.5% and 3.0–5.8% of TSP and TMA determined with three extractions. Accordingly, for untargeted LC-Q/TOF analysis, three sequential extractions were performed on three independent aliquots of each raspberry cultivar and the resulting extracts were combined. The organic solvent was removed by vacuum evaporation and filtered at 0.2 μm using nylon membranes, and the resulting aqueous extract was analyzed.

### 2.3. Pomological analyses

Colorimetric coordinates (i.e., L, a, b), total soluble solids (TSS) and titratable acids (TA) were performed according to procedures elsewhere reported [27,28], in order to evaluate the stage of maturity of the harvested fruits. In detail, colorimetric coordinates were determined with a Minolta Chromameter CR200 (Konica Minolta, Tokyo, Japan) electronic colorimeter equipped with a pulsed xenon arc lamp inside a mixing chamber, which provides diffuse, uniform lighting over the 8-mm-diameter specimen area. TSS were determined using an Atago N1 digital refractometer (Atago Co., Ltd., Tokyo, Japan) and expressed as a percentage (°Brix). TA were determined by automatic titration (877 Titrino plus, Metrohm) with 0.1 M solution of NaOH up to pH 8.1, and the results were expressed as g of citric acid (CA) per kg of fresh fruit (f.w.). The results obtained are reported in Table 1.

### 2.4. LC-Q/TOF Analysis

LC analysis was performed on an ExionLC analytical UHPLC system (SCIEX, Framingham, MA, USA) equipped with an Acquity BEH C18 column (15 cm × 2.1 cm i.d., particle size 1.7 μm) and a guard column containing the same stationary phase (Waters, Milford, MA, USA). Column temperature was set at 50 °C. LC-MS water (eluent A) and methanol (eluent B) solutions were used for the analyte elution, acidifying each solvent with formic acid at 0.1% and 5% (*v/v*) for negative and positive electrospray ionization (ESI), respectively. The following elution gradient was adopted: 0–3 min, isocratic 2% B; 3–35 min, linear gradient 2–100% B; and 35–38 min, isocratic 100% B. The flow rate was 450 μL/min and the injection volume was 5 μL. The LC system was coupled with a TripleTOF^®^ 6600 Q/TOF mass analyzer (SCIEX, Framingham, MA, USA) by the DuoSpray™ Source for TOF and Q/TOF analyses. The following source parameters were used during the acquisitions; (i) positive ionization: heater temperature 450 °C, Curtain Gas™ 30, nebulizing gas 55, heating gas 65 and spray voltage +5000 V; and (ii) negative ionization: heater temperature 450 °C, Curtain Gas™ 30, nebulizing gas 45, heating gas 55 and spray voltage −4500 V. Under both ionization modes, each extract was analyzed using the SWATH™ data independent acquisition protocol, which allows simultaneously acquiring TOF full scan MS and Q/TOF MS^2^ spectra with a comprehensive detection approach, i.e., virtually detecting all the analytes present in the extract and eluting under the chromatographic conditions adopted. The high-resolution TOF MS full scan experiment was carried out in the range 100–1000 Da (cycle time 200 ms), with an accumulation time of 150 ms and a collision energy of 70 eV. Automated calibration was performed by an external calibrant delivery system (CDS), infusing proper calibration solution prior to sample introduction.

### 2.5. Data Processing for Feature Selection and Identification

The number of raw data derived from the SWATH™ analysis of the investigated samples is very high, thus needing to be processed with specific software. In this study, Marker View^®^ 1.3.1 software was used for instrumental noise removal and blank subtraction, spectra deconvolution and chromatogram alignment, based on the TOF exact mass and isotope pattern determinations, as well as on the Q/TOF fragmentation spectra of parent ions. The following alignment criteria were adopted within the three independent samples of each cultivar: (i) TOF accuracy of the pseudo-molecular ion < 5 ppm; (ii) isotope ratio difference compared to the theoretical isotope profile < 20%; (iii) purity score of the MS^2^ spectra compared to the one of available standards ≥ 80%; and (iv) retention time (t_R_) tolerance ≤ 0.05 min [1]. Afterwards, the following workflow was adopted using the R software (version 4.0.3, https://cran.r-project.org, accessed on 1 March 2021) for the selection of a restricted group of the aligned features. In detail, the following were selected: (i) features with a coefficient of variation (CV) among cultivars higher than that in QCs; (ii) features that after the Kruskal–Wallis comparison among cultivars were significantly different at the probability level of 2% (*p*-value < 0.02); and (iii) features that after the post-hoc Dwass, Steel, Critchlow-Fligner (DSCF) multiple comparison analysis, based on pairwise two-sample Wilcoxon comparisons [29] among sample groups were significantly different at the probability level of 20% (*p*-value < 0.2). Manual revision of the features resulting from the aforementioned workflow was finally carried out with the purpose of retaining only the features providing acceptable spectral data for the successive annotation.

In this study, according to metabolomics guidelines, four levels of feature annotation were distinguished [30]. Briefly, Level I was assigned when the feature in the aligned chromatogram was successfully compared with the reference standard. For Level II annotation, the identification is performed based upon exact mass value, isotopic profile, MS^2^ spectra and chromatographic behavior of the aligned feature in comparison with internal and/or freely available external libraries (i.e., MassBank, GNPS, MetaboBASE, Fiehn/Vaniya natural product library and BMDMS-NP) of mass spectra and literature information (putatively annotated compound). The identification at Level III is based on characteristic physicochemical properties of a chemical class of compounds, or by spectral similarity to known compounds of a chemical class (putatively characterized compound classes). Finally, Level IV includes the unknown compounds.

### 2.6. Chemometrics Analyses for Genotype Comparison

To compare the metabolome profiles of the four investigated cultivars and to highlight the features that mainly contributed to their differentiation, principal component analysis (PCA) of molecular or quasi-molecular ions of the assigned compounds was performed using MarkerView software. This approach was carried out separately for compounds detected under negative and positive ionization modes. Quality control (QC) of PCA was performed, using QC samples (*n* = 3), which consisted of a mixture of equal aliquots of each raspberry extract. QC evaluation was carried out by verifying if PCA object scores obtained by replicated injections of the QC sample were close to the origin of PCA coordinates.

Pearson product-moment correlation (PPMC) analysis was also performed to evaluate the grade of correlation among the selected features and pomological data.

## 3. Results and Discussion

### 3.1. Pomological Parameters

Pomological parameters are illustrated in Table 1. The CIELAB spatial coordinates measured on raspberry skin were in the ranges of 25–53, 2–23 and 3–34 for brightness (L), green to red (a) and blue to yellow (b) axes, respectively. These very wide variations obviously reflected the different skin colors of the investigated berries. TSS and TA showed not negligible variations, being in the ranges 8.1–13.1° Brix and 10.7–19.9 g of citric acid per kg of fresh weight fruit. However, the two red raspberry cultivars (*R. idaeus* GA and T) exhibited the same CIELAB coordinates; moreover, for these two genotypes, statistically comparable values of TSS and TA were found, thus highlighting for these fruits the same stage of maturity. It should also be noted that the values of pomological parameters found here for GA, T, G and J were in quite good agreement with data previously reported for fully mature raspberries of the same cultivars [31,32,33,34] and/or for other genotypes [35], thus indicating that the harvest time was properly chosen.

### 3.2. Feature Selection and Annotation

Data peak picking and retention time alignment resulted in a very large number of features in both negative and positive ionization modes, i.e., 13,211 and 44,251, respectively. The further workflow of feature selection provided an additional restriction of the aligned data, which accounted for 10,323 and 43,774 for ESI(−) and ESI(+), respectively. Non-parametric Kruskal–Wallis test highlighted 1732 features in negative polarity and 3088 features in positive polarity. Finally, contrast analysis and manual revision of the significant features resulted in 49 and 19 features in negative and positive ionization, respectively.

All selected features were annotated according to the identification criteria reported in Section 2.5. For each feature, Table 2 and Table 3 illustrate t_R_ (min), mass to charge ratio (*m*/*z*) of the pseudo-molecular or the molecular ion found by the full scan experiment, main mass fragments, proposed formula and the corresponding exact mass, the mass accuracy (Δ, ppm), the raspberry variety in which the metabolite was identified and the tentative identification.

#### Feature Annotation

Phenolic acids: Several phenolic acids belonging to the class of the hydroxybenzoic (Peaks **1** and **2**) and hydroxycinnamic acids (**Peak**s **6**, **9**, **17**, **18** and **27**) were identified under negative ionization in at least one raspberry sample (Table 2). In detail, Peaks **1** and **2**, which were identified at Level I as gallic acid and 3,4-dihydroxybenzoic acid, respectively, were detected only in the *R. occidentalis* cultivar. Among hydroxycinnamic acids, chlorogenic acid (Peak **17**) was annotated at Level I, whereas the other acids (i.e., ferulic, *p*-coumaric and sinapic), occurring only as hexose glycosides, were identified at Level II with |Δ| of 2.4–4.9 ppm. Interestingly, the two ferulic acid hexosides identified here in *R. idaeus* fruits (Peaks **6** and **9**) represent the first report of ferulic acid glycosides in raspberry. As illustrated in Figure S1 and Scheme S1, these peaks were identified at Level II through their [M−H]^−^ pseudo-molecular ion at *m/z* 355.10, which fragmented, giving rise to peaks at *m/z* 191.02 (164 Da, loss of hexose), 147.03 and 129.02 (successive losses of CO_2_ and water, respectively), as already reported in other berries [1]. Peak **18** showed a [M−H]^−^ pseudo-molecular ion at *m/z* 325.09, which fragmented in *m/z* 118.04 and 117.03 due to the cleavage of the anomeric bond (loss of 163 Da) and successive loss of CO_2_ (loss of 44 Da). Based on these findings and previously reported patterns of fragmentation and related annotations [18,23], this feature was putatively identified (i.e., Level II) as a *p*-coumaroyl hexoside. Peak **27** with [M−H]^−^ at *m/z* 385.12 was putatively annotated as sinapic acid hexoside since it fragmented giving rise to *m/z* 223.06 (loss of the hexose group), which further fragmented in *m/z* 205.05 (formal loss of H_2_O, 18 Da) and 190.03 (loss of methyl group) (Figure S2 and Scheme S2).

**Figure 1:**
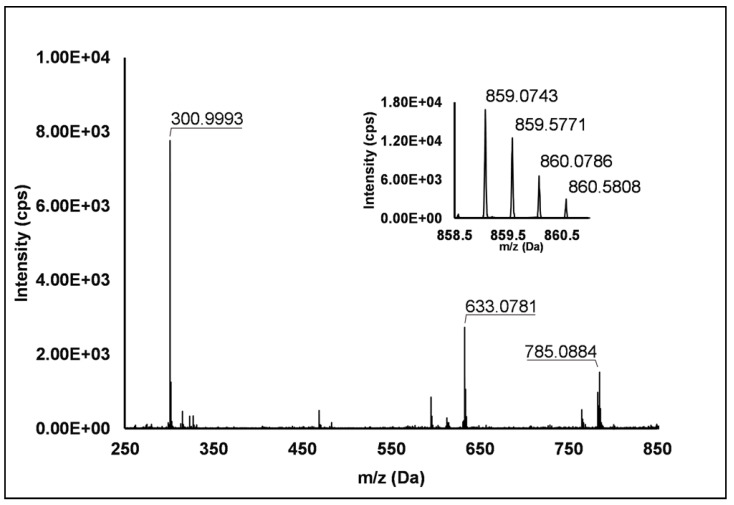
TOF MS (top right) and Q/TOF MS2 spectra of Peak **28**, tentatively identified as ellagitannin-like Nobotanin/Malabathrin.

Ellagitannins: Thirteen ellagitannins (Peaks **3**, **7**, **8**, **12**, **19**, **21**–**24** and **29**) were identified under negative ionization in both *R. idaeus* and *R. occidentalis* raspberry (Table 2). Peak **3** evidenced a [M–H]^–^ ion at *m/z* 633.07 and principal MS^2^ fragment at *m/z* 301.00 (i.e., ellagic acid), corresponding to the neutral loss of one galloyl-hexose unit. Another less intense dissociation product of the quasi-molecular ion was 331.07 (i.e., galloyl hexose), due to the loss of one hexahydroxydiphenoyl (HHDP) unit. This peak was therefore attributed to a galloyl-HHDP-hexose (Δ = 0.3 ppm), which has been previously reported in other fruit species [36,37] but not in raspberry. Peaks **7** and **19** showed double-charged ion with [M–2H]^2–^/2 at *m/z* 783.07, corresponding to a MW of 1569 Da and mono-charged fragment ions at *m/z* 935.09, 933.07, 633.08, 617.04, 331.07 and 300.99. This fragmentation pattern was in accordance with the ones observed by Zhang et al. [23] and Mullen et al. [38] in raspberry and attributed to two isomers of sanguiin H-10. Peaks **22** and **23** also showed a double-charged pseudo-molecular ion at *m/z* 934.08, corresponding to a MW of 1869 Da. The fragmentation gave rise to mono-charged ions at *m/z* 915.06, consistent with the losses of a galloyl-di-HHDP-hexose (934 Da), H_2_O and H_2_; a further loss of H_2_O originated the fragment at *m/z* 897.05. The product ions at *m/z* 633.08 (galloyl-HHDP-hexose) and 301.01 (ellagic acid) were the result of the fragmentation of the pseudo-molecular ion in the mono-charged fragment at *m/z* 934.07 (not detected, probably due to the high abundance of the double-charged ion) and its successive cleavage. Accordingly, these peaks were attributed to sanguiin H-6 isomers, already annotated in *R. idaeus* [23,39]. Peak **8** showed a double-charged ion with [M–2H]^2–^/2 at *m/z* 858.07, corresponding to a MW of 1717 Da. The fragmentation pattern included the product ions at *m/z* 935.09, 858.08, 633.08, 631.06, and 301.00, which are consistent with a sanguiin H-6 degalloylated, previously annotated by different authors in *R. idaeus* cultivars [23,39]. Peak **12** showed a mono-charged pseudo-molecular ion at *m/z* 633.08 which fragmented only in *m/z* 301.00. This peak was tentatively attributed (Level II) to corilagin, a low molecular weight ellagitannins previously identified in *R. idaeus* berries [23]. Peak **21** had a pseudo-molecular triple-charged ion at *m/z* 933.74, equivalent to a MW of 2805 Da. This peak fragmented in a double-charged ion at *m/z* 617.04 (corresponding to the loss of a di-HHDP-hexose-galloyl-HHDP) and a mono-charged ellagic acid ion (*m/z* 301.00). Based on these findings, this peak was annotated as lambertianin C, elsewhere described in *R. idaeus* berries [23,39,40]. Peak **24** was characterized by a double-charged pseudo-molecular ion at *m/z* 551.04 (corresponding to a MW of 1104 Da) and fragments at *m/z* 469.01 (HHDP-hexose), 301.00 (ellagic acid) and 169.01 (gallic acid), thus being identified as a sanguiin H-2, in accordance with a previous annotation on *R. idaeus* fruits [23]. Peak **28** exhibited a double-charged pseudo-molecular ion at *m/z* 859.08, corresponding to a MW = 1721.2 Da and fragments at *m/z* 785.09, 633.08 and 301.00 (**Figure 1**). This fragmentation pattern is consistent with the occurrence of an ellagitannin-like nobotanin/malabathrin molecule (see **Scheme S3**), which is here annotated in raspberry for the first time.

**Figure 2:**
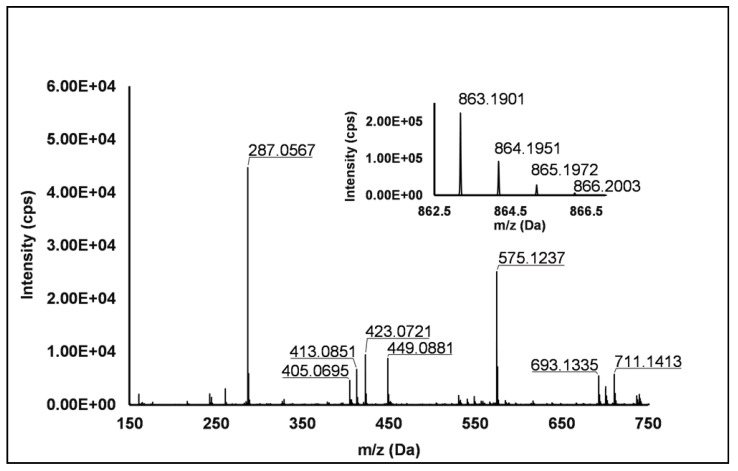
TOF MS (top right) and Q/TOF MS2 spectra of Peak **11**, tentatively identified as A/B type procyanidin trimer.

Flavanols: Eight flavanols (Peaks **4**, **5**, **10**, **11**, **14**, **16**, **20** and **25**) were identified under negative polarity in all genotypes, with the only exception of Peak **11**, which was absent in *R. occidentalis*. Flavanols occurred mainly as proanthocyanidin dimers and trimers, whereas only (+)-catechin (Peak **10**) and (−)-epicatechin (Peak **25**) were annotated (Level I) as monomers. Peaks **4**, **14** and **20** exhibited the same [M−H]^−^ quasi-molecular ion (*m/z* 577.14) and typical fragmentation of B-type (epi)catechin-(epi)catechin dimers, consisting in the retro-Diels–Alder fission of the “C” ring (*m/z* 425.09), successive loss of water (*m/z* 407.08), the cleavage of the B-type linkage with formation of the (epi)catechin monomer (*m/z* 289.07) and the fission of the heterocyclic ring of the monomer (*m/z* 125.02) [1]. Peaks **14** and **20** were undoubtedly attributed to procyanidin B1 and procyanidin B2, respectively, based on the identity confirmation with authentic standards. Accordingly, Peak **4** was putatively ascribed to a B-type procyanidin isomer, in which the C4→C6 interflavanoid bond, instead of the C4→C8 one, is present between the two (epi)catechin units. Peaks **5** and **16** showed the same pseudo-molecular ion (*m/z* 863.20) and a fragmentation pattern consistent with the presence of monomer (*m/z* 287.06) and dimer (E)C units (*m/z* 577.13 and 575.12) derived from the quinone methide reaction, thus suggesting the attribution to B-type procyanidin trimers [41]. More in detail, for Peak **5**, the undoubted attribution to procyanidin C1 was possible thanks to the availability of its authentic standard. Peak **11** showed a [M−H]^−^ pseudo-molecular ion at *m/z* 863.19 and a populated fragmentation pattern (i.e., *m/z* 711.14, 693.13, 575.12, 449.09, 423.07, 413.09 and 287.06) consistent with an A/B-linked procyanidin trimers (see Figure 2). In fact, as illustrated in Scheme S4, the product ions at *m/z* 711.14 derived from the retro Diels–Alder reaction affecting the B-type-linked (epi)catechin and gave rise to *m/z* 693.13 due to the loss of water. Moreover, *m/z* 287.06 and 575.12 were produced from the pseudo-molecular ion by the quinone methide reaction, the latter giving rise to *m/z* 449.09 and 423.08 (heterocyclic ring fissions).

Flavanols in free and conjugated forms have been scarcely investigated in raspberry samples, since only Zhang and co-workers [23] investigated this compound class in depth, even though only one red raspberry variety was investigated and few metabolites were annotated. Hence, the present study adds new knowledge on the occurrence of flavanols in raspberry.

**Figure 3:**
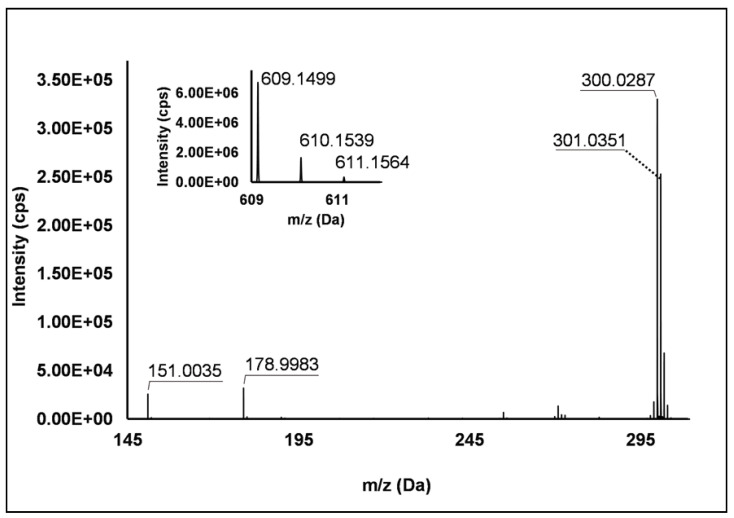
TOF MS (top left) and Q/TOF MS2 spectrum of Peak **13**, identified as quercetin-deoxyhexose-hexoside.

**Figure 4:**
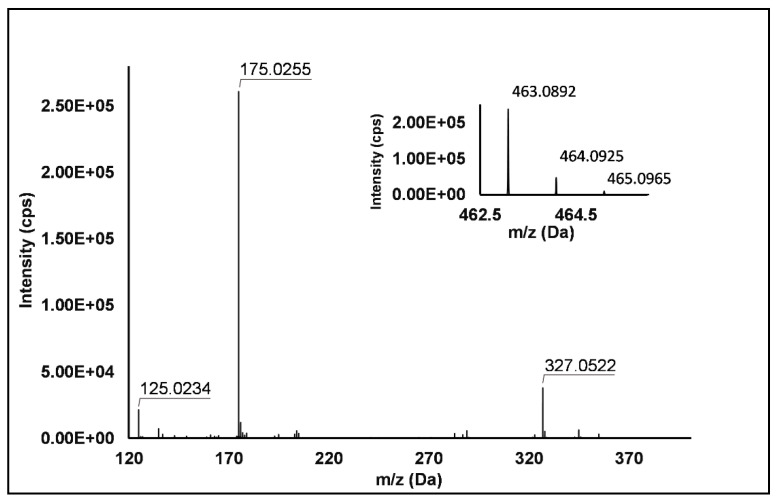
TOF MS (top right) and Q/TOF MS2 spectra of Peak **15**, tentatively identified as tetrahydroxyflavonol-3-*O*-hexoside.

**Figure 5:**
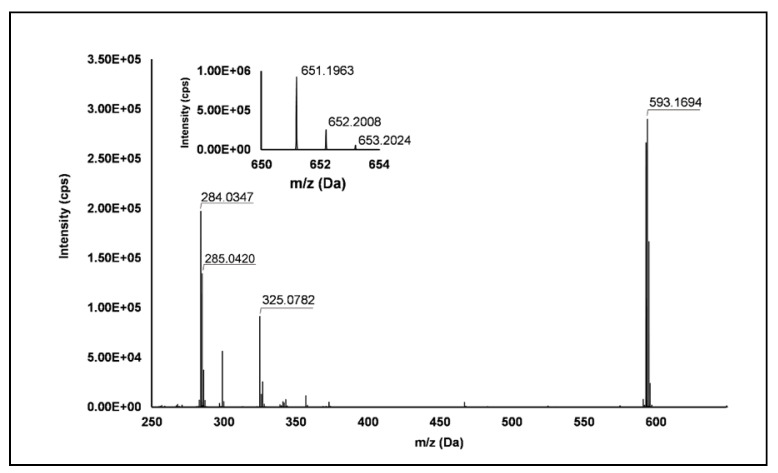
TOF MS (top left) and Q/TOF MS2 spectra of Peak **30**, tentatively identified as trihydroxy-methoxyflavone deoxyhexose-hexose derivative.

Flavonols: Nine flavonols were identified under negative polarity (Table 2), almost exclusively quercetin and kaempferol derivatives (Peaks **32**, **34**, **36–39**, **41** and **44**). Only one aglycone was found, i.e., quercetin (Peak **46**), which was identified at Level I in all the investigated cultivars, thanks to its reference standard. Authentic standards allowed the Level I identification (Table 1) of quercetin-3-*O*-sophoroside (Peak **32**) and quercetin-3,4-diglucoside (Peak **34**) in *R. idaeus*, whereas quercetin-3-*O*-galactoside (Peak **36**), quercetin-3-*O*-glucuronide (Peak **37**), quercetin-3-*O*-glucoside (Peak **38**), quercetin-3-*O*-rutinoside (Peak **39**), kaempferol-3-*O*-glucuronide (Peak **41**) and kaempferol-3-*O*-glucoside (Peak **44**) were unequivocally annotated in all the investigated raspberry samples. These annotations have been described elsewhere in *R. idaeus* fruits [18,23,42], while they represent a first report in *R. occidentalis*. The MS^2^ fragmentation pattern of these features was characterized by the heterolytic and homolytic cleavages of the glycosidic bond that produced the characteristic [Y_0_]^−^ aglycone (i.e., *m/z* 301 for quercetin and *m/z* 285 for kaempferol) and/or [Y_0_−H]^•−^ (i.e., *m/z* 300 and 284) radical aglycone ions. Peak **13**, identified only in J variety, exhibited a [M−H]^−^ pseudo-molecular ion at *m/z* 609.15, which fragmented in *m/z* 301.04 and 300.03 (Figure 3), ascribable to the [Y_0_]^−^ (Δ = −1.0 ppm) and [Y_0_−H]^•−^ (Δ = 3.7 ppm) of quercetin aglycone. This fragmentation (see Scheme S5) corresponds to the neutral loss of 308 Da, consistent with deoxyhexose-hexoside unit [1]. Further typical ions were observed at *m/z* 151.00 and *m/z* 179.00, due to different retrocyclization processes [43]. Thus, the feature was tentatively identified as quercetin-deoxyhexose-hexoside. Peak **15** exhibited a [M−H]^−^ pseudo-molecular ion at *m/z* 463.09, which is commonly found in many phenolic-rich fruits and attributed to flavonol glycosides [1,18]. However, the fragmentation pattern here observed highlighted product ions at *m/z* 327.05, 175.03 and 125.02, with no evidence of the aglycone ion formation (see Figure 4). Even though phenolic glycosides normally fragment through the cleavage of the glycosidic bond, the sugar moiety may undergo other bond breakings such as the cross-ring cleavage of the hexose part [44]. Based on the aforementioned findings, and considering the previously reported annotations of quercetin-3-*O*-hexosides (Peaks **36** and **38**), this feature was tentatively attributed to a tetrahydroxyflavonol-3-*O*-hexoside with the aglycone different from quercetin (see Scheme S6). Peak **30** exhibited a [M−H]^−^ pseudo-molecular ion at *m/z* 651.20, which fragmented giving rise to a base peak at *m/z* 593.17 and main further ions at *m/z* 325.08, 285.04 and 284.03 (Figure 5). According to the hypothesized fragmentation illustrated in Scheme S7, these latter ions are consistent with a flavonoid scaffold and can be originated from the base peak by the formal loss of a deoxyhexose-hexoside moiety, due to the homolytic (*m/z* 284.03) or heterolytic (*m/z* 285.04) fission of the glycosidic bond. Finally, the product ion at *m/z* 325.08 derived from the cleavage of the carbon-oxygen bond on the aglycone ring. Thus, such structural information led to the tentative annotation of Peak **30** to a trihydroxy-methoxyflavone deoxyhexose-hexose derivative. One flavanol (Peak **55**) was also identified under positive ionization (Table 3). In detail, this feature exhibited [M + H]^+^ pseudo-molecular ion at *m/z* 481.10, which fragmented giving rise to the loss of 162.05 Da (hexose unit) and the product ion at *m/z* 319.05, consistent with the myricetin aglycone. The comparison with t_R_ and MS^2^ fragmentation pattern of myricetin-3-*O*-glucoside reference standard confirmed the attribution to a myricetin hexoside but excluded the occurrence of the glucoside derivative. Myricetin derivatives have been identified as typical metabolomics traits of several berries [45], but this study revealed for the first time their occurrence in *R. idaeus* yellow variety.

Anthocyanins: As illustrated in Table 3, the features identified in positive ionization mode were mostly anthocyanins, which were annotated at Levels I and II. In detail, the majority of the anthocyanins detected in the four raspberry varieties were cyanidin mono-, di- and tri-glycosides. These antioxidant compounds are well-known metabolic traits of raspberries, and in general of berries, being responsible for their red to purple pigmentation [19,46]. Peaks **50**, **52**, **54**, **57** and **63** were annotated at Level I as cyanidin-3-*O*-sophoroside, cyanidin-3-*O*-galactoside, cyanidin-3-*O*-glucoside, cyanidin-3-*O*-rutinoside and cyanidin-3-*O*-arabinoside, respectively. These anthocyanins occurred in all the investigated raspberry fruit samples (intensity order: J >> T > GA > G), with the only exception of Peak **63**, which was found only in *R. occidentalis* variety. Other cyanidin derivatives, elsewhere annotated in *Rubus* species [17,18,23,47], were identified here throughout the investigated samples. Among them, Peaks **53**, **60** and **64** were annotated at Level II in GA, T and J cultivars, while they were absent in G variety. Peak **53** exhibited [M]^+^ molecular ion at *m/z* 727.21 and MS^2^ spectra highlighting successive losses of 146 Da (deoxyhexose unit) up to the formation of the well-known cyanidin aglycone at *m/z* 287.06. Thus, Peak **53** was putatively annotated as cyanidin-3-*O*-xylosilrutinoside, which was previously reported in *R. idaeus* berries [18]. Peaks **60** and **64** exhibited the same [M]^+^ ion at *m/z* 595.17 and fragmentation pattern, which was characterized by successive losses of deoxyhexose (146 Da) and hexose (162 Da) units, giving rise to the cyanidin aglycone. These peaks were putatively annotated as two positional isomers of cyanidin diglycoside with hexose and rhamnose units. This attribution was consistent with: (i) their increased hydrophobicity compared to Peak **57** (cyanidin-3-*O*-rutinoside), ascribable to different linkages of the two glycosidic moieties (e.g., C3−glucose and C7−rhamnose) [48]; and (ii) the matches of their MS^2^ spectra with that of Peak **57**. Peak **62** showed a unique loss (132 Da) in its MS^2^ spectra, leading to cyanidin aglycone fragment, which was ascribable to aldopentose loss [1]; moreover, thanks to the spectral matches with cyanidin-3-*O*-arabinoside reference standard, it was addressed at Level II as cyanidin-3-*O*-aldopentose. The remaining cyanidin derivatives were identified at Level II as cyanidin-3-*O*-(2G-glucosylrutinoside) (Peak **51**, Δ = 0.1 ppm) and cyanidin-3-*O*-malonyl-glucoside (Peak **68**, Δ = −1.5 ppm) with high mass accuracy, according to the isotopic profile of molecular ions and fragmentation pattern reported in previous studies [1,23].

Other fundamental anthocyanins in raspberries were pelargonidin derivatives, being their occurrence strongly dependent on fruit varieties [2], generally following the order J > T ≥ GA >> G. In detail, pelargonidin glycosides (Table 3) were putatively identified through the presence of the aglycone peak in the MS^2^ spectra (*m/z* 271.06) and of specific losses [18,23]: Peak **56** is the sophorose loss (324 Da,), Peak **58** is the successive losses of deoxyhexose (146 Da) and rutinose (324 Da) moieties, Peak **59** is the loss of glucose (162 Da) and Peak **61** is the deoxyhexose loss followed by glucose loss. Peak **56** occurred in the entire set of investigated raspberry cultivars, whereas Peaks **58** and **59** were both absent in yellow fruits, in which the anthocyanin content is strongly reduced [42]. As expected, pelargonidin-3-*O*-rutinoside was detected only in *R. occidentalis* black raspberries, since it has been already recognized as a specific trait of these fruits [2,16]. Thanks to the availability of reference standards, other two anthocyanins were identified at Level I in the four raspberry varieties; in detail, Peaks **65** and **66** were addressed as peonidin-3-*O*-glucoside and delphinidin-3-*O*-rutinoside, respectively. The former compound was detected in all investigated samples, notably for the very first time in yellow cultivar, while the latter occurred only in *R. occidentalis* fruits. It should be noted that delphinidin derivatives have been generally associated to the secondary metabolism of bilberry, blueberry and blackcurrant [1,49], and they represent an uncommon trait for *R. idaeus* species, but they can occur in different raspberry species [50] as reported in this study. Overall, the occurrence profile of anthocyanins here presented (i.e., J > T ≥ GA > G) agreed with previously reported data on raspberries. In fact, the content of anthocyanins in black cultivars is generally 3–10 times higher than in red fruits [46,51].

Other phenolics: Under negative ionization, nine further phenolic compounds (i.e., Peaks **26**, **29**, **31**, **33**, **35**, **40**, **42**, **43**, **45** and **47**) were putatively or unequivocally identified in at least one of the four raspberry varieties investigated (Table 2). Peak **26** (detected only in G *Rubus idaeus* fruits) was characterized by a mono-charged precursor ion at *m/z* 341.12, which fragmented in *m/z* 179.07 and 121.03. The ion at *m/z* 341.12 derived from the loss of 162 Da (hexose unit) from the quasi-molecular ion and further fragmented in *m/z* 121.03 (loss of 57.03 Da). Interestingly, the two fragments are consistent with mass spectra of the available standard of coniferyl alcohol and literature findings [52]; accordingly, Peak **26** was undoubtedly identified as coniferin. This compound was previously annotated in the yellow raspberry cultivar “Heritage” and can therefore considered as a typical metabolic trait of yellow *R. idaeus* fruits [42]. Peaks **31**, **35**, **40**, **43** and **47** were identified at Level I as taxifolin (flavanolol), ellagic acid, naringenin-7-*O*-glucoside (flavanone), phloridzin and phloretin (chalcones), confirming the annotations previously reported in raspberry varieties [23,25]. Peak **33** was also annotated at Level I and ascribed to polydatin (i.e., trihydroxystilbene-3-*O*-glucoside), the occurrence of which is conversely reported here for the first time in *R. occidentalis* berries. Peak **29** exhibited a [M–H]^–^ pseudo-molecular ion at *m/z* 593.15, and it was putatively annotated with good accuracy (Δ = −0.1 ppm) as apigenin diglucoside in comparison with spectral libraries. In fact, its fragmentation originated the product ions at *m/z* 475.14 (loss of 118 Da, retro-cyclization of the flavonoid C ring) and at *m/z* 431.06 (loss of 162 Da, consistent with a hexoside moiety). The latter further fragmented giving rise to the base peak at *m/z* 245.14, deriving from a retro-cyclization process and a consequent loss of 72 Da. Peaks **42** ([M−H]^–^ = 447.06 Da) and **45** ([M−H]^–^ = 475.05 Da) were preliminary recognized owing to the occurrence of fragments at *m/z* 315.02 and 301.00 (base peaks), which corresponded with good accuracy to methylellagic acid (Δ = 18.7 ppm) and ellagic acid (Δ = −3.3 ppm) units. The pseudo-molecular ion of Peak **42** loosed a pentoside group (i.e., 132 Da) giving rise to the aforementioned base peak, which further fragmented in *m/z* 285.04, consistently with the loss of a methoxy group (31 Da). Peak **42** was therefore putatively identified as methylellagic acid pentose conjugate. Instead, the precursor ion of Peak **45**, besides base peak, was characterized by fragments at *m/z* 432.03 and 329.13, originated from a loss of 43 Da (acetyl moiety) and from the cross-ring cleavage of the saccharide moiety, respectively. Based on these considerations and on findings previously reported in the literature [53], this peak was tentatively annotated as ellagic acid acetyl-pentose conjugate.

Non-phenolic compounds: Among the features identified in negative ionization (Table 2), Peaks **48** ([M−H]^−^ = 503.34 Da) and 49 ([M−H]^−^ = 487.34 Da) were detected in at least two raspberry varieties and attributed to the free triterpenic acids (TTPAs) madecassic acid (Δ = 3.6 ppm) and asiatic acid (Δ = −0.4 ppm). The two peaks exhibited similar MS^2^ fragmentation patterns, consistent to the aforementioned annotations, owing to successive neutral losses of water (18 Da) and CO_2_ (44 Da). The isotopic profile of full scan MS and MS^2^ spectra were also consistent with TTPAs [54]. The Level II identification of asiatic acid (Peaks **49**) in T and GA varieties was in agreement with findings previously reported [55], while madecassic acid (Peak **48**) was detected here for the first time in *R. idaeus* varieties.

**Figure 6:**
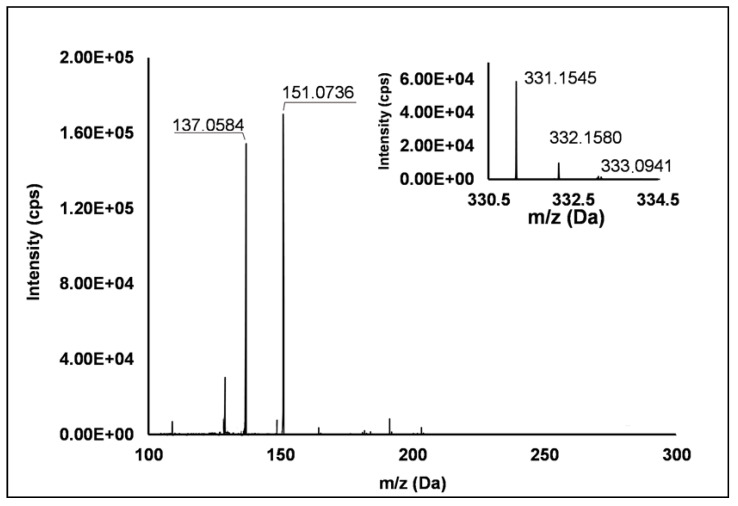
TOF MS (top right) and Q/TOF MS2 spectra of Peak **67**, tentatively identified as gibberellin A7.

Among the set of features identified in positive ionization mode (Table 3), Peak **67** showed a [M + H]^+^ pseudo-molecular ion at *m/z* 331.15 (Δ = −0.3 ppm), which occurred in all the raspberry sample here investigated. The fragmentation pattern (Figure 6) was characterized by the cleavage of the five-carbon ring, which gave rise to *m/z* 151.07, and the successive loss of the methyl group producing *m/z* 137.06 (see Scheme S8). Accordingly, thanks to the analysis of available spectral libraries, Peak **67** was putatively ascribed to gibberellin A7. Although gibberellins have been already investigated as phytohormones responsible of flowering in *R. idaeus* plants [56,57,58], this study provided for the first time its occurrence in *R. occidentalis* J variety.

### 3.3. Genotype Effects

A multivariate elaboration of the autoscaled original data (e.g., chromatographic feature intensities) was performed separately for compounds detected in negative and positive polarity, by means of PCA in order to discover possible clusterization among the investigated samples. As regards compounds detected in negative polarity, four principal components (PCs), characterized by eigenvalues > 1 and accounting for percentages of explained variances (EV%) of 55.4%, 19.5%, 13.1% and 5.2%, were obtained (total EV% = 93.2). Four significant PCs were also found for features identified in positive ionization, each one accounting for EV% of 55.2%, 16.6%, 13.6% and 8.4% (total EV% = 93.8%).

**Figure 7:**
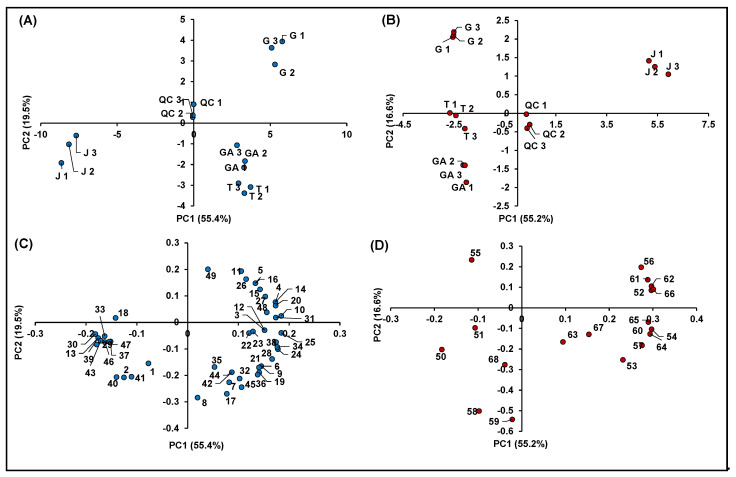
Principal component analysis of the 68 identified significant features in *R. idaeus* and *R. occidentalis* fruits. (**A**) Score plot PC1 vs. PC2 of identified features in negative ionization; (**B**) score plot PC1 vs. PC2 of identified features in positive ionization; (**C**) loading plot PC1 vs. PC2 of identified features in negative ionization; and (**D**) loading plot PC1 vs. PC2 of identified features in positive ionization.

Figure 7 summarizes the information obtained from the metabolomics profiling of the native compounds occurring in the investigated raspberry samples (including QCs), allowing for identifying more easily possible genotype effects: (i) among different cultivars of *R. idaeus;* and (ii) between *R. idaeus* and *R. occidentalis* species. In detail, Figure 7A,B illustrates the score plots of PC1 vs. PC2 of the significant features identified in negative and positive polarity, respectively, while Figure 7C,D shows the corresponding loading plots. In both datasets, the percentage of cumulative EV% of PC1 vs. PC2 space was > 70%, making the PCA results shown in Figure 7 highly significant. Moreover, in both score plots, QCs were very close to the origin of the coordinates, indicating the high accuracy and precision of the entire analytical procedure. It should also be remarked that, in both score graphs, replicated samples showed very similar coordinates, thus indicating the homogeneous results obtained within each treatment.

Both score plots showed a strong discrimination among different cultivars of *R. idaeus* and also between *R. idaeus* and *R. occidentalis* species, thus highlighting an effect of genotype on the expression of the annotated (poly)phenols. Interestingly, the four cultivars clustered according to their color not only as a function of their anthocyanin content (Figure 7B), but also in terms of the expression of other (poly)phenolics (Figure 7A). More in detail, as illustrated in Figure 7C, the red raspberries (i.e., GA and T) were mostly represented by ellagic acid and its derivatives and especially ellagitannins, which occurred in these cultivars with much higher intensities compared to J and G. Conversely, the G variety (*R. idaeus*) was mainly discriminated by the remarkable occurrence of flavanols, while the clusterization of the J cultivar (*R. occidentalis*) was due to the predominant presence of a miscellaneous compounds, which included mainly some flavonols and phenolic acids, one stilbene and two chalcones. A clearer scenario was highlighted for anthocyanins (Figure 7D), which were by far more abundant in J (*R. occidentalis*) as expected by its colorimetric analysis (Table 1). Cyanidin-3-*O*-sophoroside (Peak **50**) and cyanidin-3-*O*-(2G-glucosylrutinoside) (Peak **51**) were the only exceptions, being them more expressed in GA and T (*R. idaeus*).

### 3.4. Correlations Between Identified Features and Pomological Parameters

**Figure 8:**
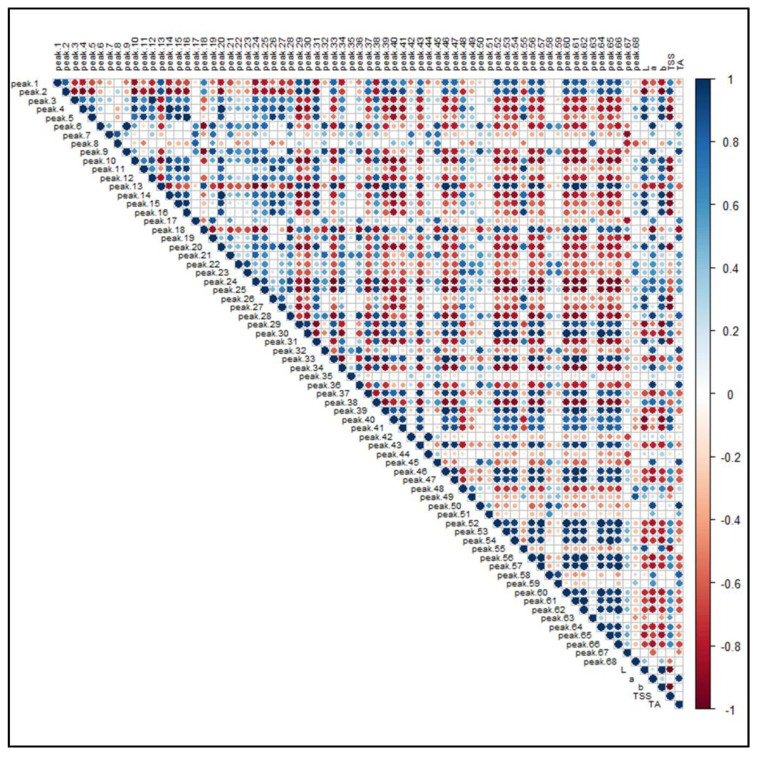
Pearson product-moment correlation analysis of the 68 identified features in *R. idaeus* and *R. occidentalis* berries against pomological parameters. Features are indexed as reported in Table 1 (T1) and Table 2 (T2), respectively. Standard pomological data are reported in Table 1. L, brightness; a, red-shift; b, yellow-shift; TSS, total soluble solids (°Brix); TA, titratable acids (g citric acid kg^−1^ f.w.). The colored scale indicates the degree of correlation (white to blue color) or anti-correlation (white to red color).

Figure 8 illustrates the PPMC analysis of the features identified in both ionization modes in relation to the main pomological parameters (Table 1) of the investigated raspberries. A significant (*p*-value < 0.05) positive correlation was found between brightness (L) and the presence of free and condensed flavanols (i.e., Peaks **4**, **5**, **10**, **16**, **20** and **26**), since these metabolites were found to be a distinctive metabolic trait of the yellow and brighter cultivar G, as observed in the PCA. A positive correlation, although characterized by a greater coefficient spread, was also observed between ellagitannins (i.e., Peaks **7**, **8**, **12**, **19**, **21–24** and **28**) and some phenolic acid glycosides (i.e., Peaks **6**, **9**, **18** and **27**). Moreover, the signal intensity of both these groups of compounds positively correlated with the “a” color coordinate, indicating that, as the fruit color turns red, the intensities of these (poly)phenols increased. Conversely, some anthocyanins exhibited an unexpected statistically significant inverse correlation with respect to the coordinate “a”. However, the predominant anthocyanins of raspberries, i.e., the Peaks **50**, **51**, **58** and **59**, were strongly and significantly correlated with the red color of fruits. Interestingly, a positive correlation was found among anthocyanins, flavonols glycosides and TSS, suggesting that the expression of soluble sugars, which contribute extensively to this parameter, is related to the presence of some (poly)phenolic classes. As a final outcome, the acidity of raspberry fruits, expressed as titratable acids (TA), is significantly correlated to the presence of hydroxycinnamic acids (i.e., Peaks **6**, **9** and **18**), ellagitannins (i.e., Peaks **7**, **8**, **12**, **19**, **21–24** and **28**) and ellagic acid and its derivatives (i.e., Peaks **35**, **42** and **45**).

## 4. Conclusions

This paper offers novel information on the (poly)phenolic composition of raspberry fruits, thanks to the adopted comprehensive untargeted strategy and data processing for features annotation, together with identity confirmation with authentic standards.

Briefly, 68 bioactive compounds were successfully identified at Levels I and II, providing an in-depth characterization of the investigated raspberry genotypes. Even though most of the identified features belong to the already known major categories of (poly)phenols occurring in raspberry fruits (i.e., phenolic acids, ellagitannins, flavonols, flavanols and anthocyanins), this study extends the current knowledge of native composition on *R. idaeus* and above all *R. occidentalis*, the latter being scarcely investigated elsewhere. In fact, ferulic acid glycosides, one ellagitannin and madecassic acid were annotated for the first time in all the four investigated varieties. Additionally, the annotation of flavonol glycosides, polydatin and gibberellin A7 in *R. occidentalis* and of myricetin hexoside and peonidin-3-glucoside in the G raspberry cultivar of the *R. idaeus* species represent a first report in the investigated samples.

The identification of few non-phenolic compounds possessing antioxidant properties, such as triterpenic acids, suggested the importance of extending untargeted investigations to compounds other than (poly)phenolic compounds.

Overall, the in-depth profiling of raspberry secondary metabolites, together with the PCA carried out on the identified features, clearly highlighted the presence of genotype effects: (i) among different cultivars of *R. idaeus*; and (ii) between *R. idaeus* and *R. occidentalis* species. The latter represents an aspect investigated here for the first time.

## Figures and Tables

**Table 1:** Fruit pomological characterization of the investigated raspberry samples. Values of colorimetric coordinates (L, a and b), total soluble solids (TSS, expressed as Brix) and titratable acids (TA) are reported as means (*n* = 3) and relative standard deviations (in brackets). GA, Glen Ample; G, Fall Gold; T, Tulameen; J, Jewel; CA, citric acid; f.w., fruit fresh weight. Values marked with a different superscript letter (a, b, c) are statistically different (*p*-value < 0.05) according to Kruskal–Wallis Test.

	L	a	b	TSS (°Brix)	TA (g CA kg^−1^ f.w.)
***Rubus idaeus***					
**GA**	35 (1) ^a^	23 (3) ^a^	16 (3) ^a^	11.1 (1.1) ^a^	19.7 (1.4) ^a^
**T**	35 (3) ^a^	23 (3) ^a^	16 (3) ^a^	11.8 (1.7) ^a^	19.9 (1.3) ^a^
**G**	53 (1) ^b^	12 (2) ^a^	34 (2) ^b^	8.1 (1.1) ^b^	12.4 (1.0) ^b^
***Rubus occidentalis***					
**J**	25 (1) ^c^	2 (1) ^b^	3 (1) ^c^	13.1 (1.1) ^c^	10.7 (1.0) ^c^

**Table 2:** Retention times (t_R_, min), pseudo-molecular ions (TOF, Da), mass fragments (Q/TOF, Da), proposed formula, corresponding exact mass (Da) and accuracy (Δ, ppm) of peaks tentatively identified under negative ionization. Symbols + and − mean detected and not detected, respectively. GA, Glen Ample; T, Tulameen; J, Jewel; G, Fall Gold. ^a^ Peaks annotated at Level I. The base peak of each MS^2^ spectra is reported in bold character. Fragments marked with asterisk are double-charged ions.

Peak	t_R_	TOF	Charge	Q/TOF	Formula	Exact Mass	Δ	GA	T	G	J	Tentative Identification
1	1.61	169.0144	−H	**125.0233**; 126.0275; 124.0154; 79.0173	C_7_H_6_O_5_	169.0143	0.6	−	−	−	+	Gallic acid ^a^
2	3.17	153.0200	−H	**109.0291**; 108.0210	C_7_H_6_O_4_	153.0194	3.9	−	−	−	+	3,4-Dihydroxybenzoic acid ^a^
3	5.28	633.0735	−H	331.0685; **301.0003**	C_27_H_22_O_18_	633.0733	0.3	+	+	+	+	Galloyl-HHDP-hexose
4	6.03	577.1380	−H	425.0909; **407.0804**; 289.0732; 125.0232	C_30_H_26_O_12_	577.1352	4.9	+	+	+	+	B-type procyanidin dimer
5	6.26	865.2017	−H	**577.1394**; 575.1259; 287.0570	C_45_H_38_O_18_	865.1985	3.7	+	+	+	+	Procyanidin C1 ^a^
6	6.34	355.1024	−H	191.0203; 147.0304; **129.0192**	C_16_H_20_O_9_	355.1035	−3.1	+	+	+	−	Ferulic acid hexoside I
7	6.49	783.0703	−2H	935.0892; 933.0731; 633.0777; 617.0370; 331.0679; **300.9993**	C_68_H_48_O_44_	783.0687	2.0	+	+	+	+	Sanguiin H-10 I
8	6.68	858.0684	−2H	935.0890; 858.0752; 633.0756; 631.0607; **300.9991**	C_75_H_50_O_48_	858.0663	2.4	+	+	+	+	Sanguiin H-6 degalloylated
9	6.89	355.1026	−H	191.0203; 147.0304; **129.0192**	C_16_H_20_O_9_	355.1035	−2.5	+	+	+	−	Ferulic acid hexoside II
10	7.06	289.0724	−H	245.0819; 205.0502; 203.0707; **125.0228**; 123.0434; 109.0276	C_15_H_14_O_6_	289.0718	2.2	+	+	+	+	(+)-Catechin ^a^
11	7.13	863.1903	−H	711.1413; 693.1335; 575.1237; 449.0896; 423.0721; 413.0851; 405.0695; **287.0567**	C_45_H_36_O_18_	863.1914	−1.3	+	+	+	-	A/B type procyanidin trimer
12	7.31	633.0760	−H	**300.9993**	C_27_H_22_O_18_	633.0733	4.3	+	+	+	+	Corilagin
13	7.31	609.1490	−H	**300.0287**; 301.0351; 178.9983; 151.0035	C_27_H_30_O_16_	609.1461	4.7	−	−	-	+	Quercetin deoxyhexose-hexoside
14	7.46	577.1378	−H	407.0822; **289.0757**; 125.0233	C_30_H_26_O_12_	577.1352	4.6	+	+	+	+	Procyanidin B1 ^a^
15	7.67	463.0892	−H	327.0522; **175.0255**; 125.0234	C_21_H_20_O_12_	463.0882	2.2	+	+	+	−	Tetrahydroxyflavonol-3-*O*-hexoside
16	7.85	865.2023	−H	**577.1380**; 407.0781; 287.0560; 125.0224	C_45_H_38_O_18_	865.1985	4.4	+	+	+	+	B-type procyanidin trimer
17	8.13	353.0884	−H	**191.0551**	C_16_H_18_O_9_	353.0878	1.7	+	+	+	+	Chlorogenic acid ^a^
18	8.37	325.0940	−H	**146.0319**; 145.0289; 118.0364; 117.0332	C_15_H_18_O_8_	325.0929	3.4	+	+	+	+	*p*-Coumaryl hexoside
19	8.44	783.0702	−2H	935.0892; 933.0731; 633.0777; 617.0370; 331.0679; **300.9993**	C_68_H_48_O_44_	783.0687	1.9	+	+	+	+	Sanguiin H-10 II
20	8.60	577.1355	−H	425.0887; 407.0775; **289.0716**	C_30_H_26_O_12_	577.1351	0.6	+	+	+	+	Procyanidin B2 ^a^
21	9.20	933.7395	−3H	617.0367 *; **300.9912**	C_123_H_80_O_78_	933.7358	4.0	+	+	+	+	Lambertianin C
22	9.25	934.0796	−2H	915.0632; 897.0499; 633.0775; **301.0077**	C_82_H_54_O_52_	934.0757	4.2	+	+	+	+	Sanguiin H-6 I
23	9.57	934.0779	−2H	915.0618; 897.0485; 633.0759; **301.0056**	C_82_H_54_O_52_	934.0737	4.5	+	+	+	+	Sanguiin H-6 II
24	9.58	551.0433	−2H	469.0072; **300.9998**; 169.0133	C_48_H_32_O_31_	551.0410	4.2	+	+	+	+	Sanguiin H-2
25	9.82	289.0724	−H	245.0818; 203.0704; 125.0226; **123.0434**	C_15_H_14_O_6_	289.0717	2.2	+	+	+	+	(−)-Epicatechin ^a^
26	10.23	341.1245	−H	**179.0710**; 121.0280	C_16_H_22_O_8_	341.1241	1.0	−	−	+	−	Coniferin
27	10.28	385.1154	−H	223.0622; **205.0528**; 190.0276	C_17_H_22_O_10_	385.1135	4.9	+	+	+	−	Sinapic acid hexoside
28	10.33	859.0802	−2H	785.0884; 633.0781; **300.9993**	C_75_H_52_O_48_	859.0760	4.9	+	+	+	−	Ellagitannin-like Nobotanin/Malabathrin
29	10.61	593.1510	−H	475.1400; 431.0600; **245.1390**	C_27_H_30_O_15_	593.1506	−0.01	+	+	−	+	Apigenin diglucoside
30	11.69	651.1983	−H	**593.1694**; 325.0782; 285.0420; 284.0347	C_30_H_38_O_17_	651.1945	4.6	+	+	−	+	Trihydroxy-methoxyflavone deoxyhexose-hexose derivative
31	11.71	303.0523	−H	285.0417; **125.0233**	C_15_H_12_O_7_	303.0510	4.2	−	−	+	−	Taxifolin ^a^
32	11.91	625.1433	−H	**301.0323**; 245.0937	C_27_H_30_O_17_	625.1410	3.7	+	+	+	−	Quercetin-3-*O*-sophoroside ^a^
33	12.14	389.1244	−H	**227.0718**	C_20_H_22_O_8_	389.1241	0.5	−	−	−	+	Polydatin ^a^
34	12.20	625.1434	−H	**301.0356**	C_27_H_30_O_17_	625.1410	3.8	+	+	+	−	Quercetin-3,4-diglucoside ^a^
35	13.89	301.0002	−H	270.9953; 257.0102; 245.0096; **229.0152**	C_14_H_6_O_8_	300.9989	4.0	+	+	+	+	Ellagic acid ^a^
36	13.97	463.0870	−H	300.0281; **301.0341**; 271.0245	C_21_H_20_O_13_	463.0882	−2.6	+	+	+	+	Quercetin-3-*O*-galactoside ^a^
37	13.99	477.0680	−H	**301.0354**; 178.9969; 151.0025	C_21_H_18_O_13_	477.06746	1.1	+	+	+	+	Quercetin-3-*O*-glucoronide ^a^
38	14.34	463.0890	−H	300.0281; **301.0341**; 271.0244	C_21_H_20_O_12_	463.0882	1.7	+	+	+	+	Quercetin-3-*O*-glucoside ^a^
39	14.39	609.1483	−H	300.0287; **301.0351**	C_27_H_30_O_16_	609.14611	3.6	+	+	+	+	Quercetin-3-*O*-rutinoside ^a^
40	14.68	433.1144	−H	**271.0606**	C_21_H_22_O_10_	433.114	0.9	+	+	+	+	Naringenin-7-*O*-glucoside ^a^
41	15.61	461.0730	−H	447.0615; 315.0188; **285.0417**	C_21_H_18_O_12_	461.07255	1.0	+	+	+	+	Kaempferol-3-*O*-glucoronide
42	15.64	447.0572	−H	**315.0205**; 285.0418	C_20_H_16_O_12_	447.0569	0.7	+	+	+	+	Methylellagic acid pentose conjugate
43	15.64	435.1302	−H	**273.0774**; 229.0868; 167.0.347	C_21_H_24_O_10_	435.12967	1.2	+	+	+	+	Phloridzin ^a^
44	15.86	447.0942	−H	300.0281; **284.0326**	C_21_H_20_O_11_	447.09329	2.0	+	+	+	+	Kaempferol-3-*O*-glucoside ^a^
45	15.97	475.0522	−H	432.0343; 329.1265; **300.9980**	C_21_H_16_O_13_	475.05181	0.8	+	+	+	−	Ellagic acid acetyl-pentose conjugate
46	17.73	301.0357	−H	**178.9970**; 151.0028; 121.0277	C_15_H_10_O_7_	301.03538	1.1	+	+	+	+	Quercetin ^a^
47	19.01	273.0776	−H	**167.0357**; 125.0227; 123.0435; 119.0487	C_15_H_14_O_5_	273.07685	2.7	−	−	−	+	Phloretin ^a^
48	26.37	503.3396	−H	**485.3325**; 441.485	C_30_H_48_O_6_	503.33781	3.6	+	+	+	−	Madecassic acid
49	27.22	487.3427	−H	**469.3341**; 425.3448	C_30_H_48_O_5_	487.3429	−0.4	+	+	−	−	Asiatic acid

**Table 3:** Retention times (t_R_, min), molecular and pseudo-molecular ions (TOF, Da), mass fragments (Q/TOF, Da), proposed formula, corresponding exact mass (Da) and accuracy (Δ, ppm) of peaks identified under positive ionization. Symbols + and − mean detected and not detected, respectively. GA, Glen Ample; T, Tulameen; J, Jewel; G, Fall Gold. ^a^ Peaks annotated at Level I. The base peak of each MS^2^ spectra is reported in bold character.

Peak	t_R_	TOF	Charge	Q/TOF	Formula	Exact Mass	Δ	GA	T	G	J	Tentative Identification
50	7.07	611.1604		**287.0574**; 449.1067	C_27_H_31_O_16_	611.1621	−2.8	+	+	+	+	Cyanidin-3-*O*-sophoroside ^a^
51	7.3	757.2191		757.1961; 611.1593; **287.0577**	C_33_H_41_O_20_	757.21912	0.1	+	+	−	+	Cyanidin-3-*O*-(2G-glucosylrutinoside)
52	7.38	449.1080		**287.062**	C_21_H_21_O_11_	449.10839	−0.8	+	+	+	+	Cyanidin-3-galactoside ^a^
53	7.39	727.2073		581.1491; 433.1129; 281.0590	C_32_H_39_O_19_	727.20855	−1.7	+	+	−	+	Cyanidin 3-xylosylrutinoside
54	7.4	449.1085		**287.0603**	C_21_H_21_O_11_	449.1084	0.2	+	+	+	+	Cyanidin-3-*O*-glucoside ^a^
55	7.44	481.0973	+H	**319.0462**	C_21_H_20_O_13_	481.0982	−1.9	−	−	+	-	Myricetin hexoside
56	7.57	595.1658		287.0626; **271.0657**	C_27_H_31_O_15_	595.1663	−0.9	+	+	+	+	Pelargonidin-3-*O*-sophoroside
57	7.64	595.1672		449.1072; **287.0689**	C_27_H_31_O_15_	595.1663	1.5	+	+	+	+	Cyanidin-3-*O*-rutinoside ^a^
58	7.8	741.2243		549.1948; **271.0644**	C_33_H_41_O_19_	741.2242	0.1	+	+	−	−	Pelargonidin-3-*O*-(2G)-glucosylrutinoside
59	7.93	433.1135		305.1562; **271.0653**	C_21_H_21_O_10_	433.11347	0.1	+	+	−	+	Pelargonidin-3-*O*-glucoside
60	8.08	595.1660		449.1058; **287.0702**	C_27_H_31_O_15_	595.1663	−0.5	+	+	−	+	Cyanidin hexoside rhamnoside I
61	8.16	579.1693		453.0077; **271.0595**	C_27_H_31_O_14_	579.17138	−3.6	−	−	−	+	Pelargonidin-3-*O*-rutinoside
62	8.25	419.0971		**287.0543**	C_20_H_19_O_10_	419.09782	−1.7	+	−	−	+	Cyanidin-3-*O*-aldopentose
63	8.26	419.0975		301.0719; **287.0552**	C_20_H_19_O_10_	419.09782	−0.7	−	−	−	+	Cyanidin-3-arabinoside ^a^
64	8.29	595.1662		449.1060; **287.0701**	C_27_H_31_O_15_	595.1663	−0.2	+	+	−	+	Cyanidin hexoside rhamnoside II
65	8.31	463.1251		**301.0731**	C_22_H_23_O_11_	463.12404	2.3	+	+	+	+	Peonidin-3-*O*-glucoside ^a^
66	9.35	611.1608		**303.0517**	C_27_H_31_O_16_	611.16121	−0.7	−	−	−	+	Delphinidin-3-*O*-rutinoside
67	10.11	331.1545	+H	287.1258; 285.1118; **151.0736**; 137.0584	C_19_H_22_O_5_	331.1540	−0.3	+	+	+	+	Gibberellin A7
68	10.88	535.1080		487.2175; **287.5045**	C_24_H_23_O_14_	535.10878	−1.5	+	−	+	+	Cyanidin-3-*O*-malonyl-glucoside

## Data Availability

The data are contained within the article or the Supplementary Materials.

## Supplementary Materials

The following are available online at https://www.mdpi.com/article/10.3390/antiox10050704/s1, Figure S1: TOF MS (top right) and Q/TOF MS2 spectra of Peaks 6 and 9 tentatively identified as ferulic acid hexosides. Scheme S1: Hypothesized structure and fragmentation scheme for Peaks 5 and 6 ([M–H]^−^ = 355.1024). Figure S2: TOF MS (top right) and Q/TOF MS2 spectra of Peak 27 tentatively identified as sinapic acid hexosides. Scheme S2: Hypothesized structure and fragmentation scheme for Peak 27 ([M–H]^−^ = 385.1154). Scheme S3: Hypothesized structure and fragmentation scheme for Peak 28 ([M–2H]^2−^/2 = 859.0802). Scheme S4: Hypothesized structure and fragmentation scheme for Peak 11 ([M–H]^−^ = 863.1903). Scheme S5: Hypothesized structure and fragmentation scheme for Peak 13 ([M–H]^−^ = 609.1499). Scheme S6: Hypothesized structure and fragmentation scheme for Peak 15 ([M–H]^−^ = 463.0892). Scheme S7: Hypothesized structure and fragmentation scheme for Peak 30 ([M–H]^−^ = 651.1963). Scheme S8: Hypothesized structure and fragmentation scheme for Peak 67 ([M + H]^+^ = 331.1545).

## References

[1] Ancillotti C., Ciofi L., Rossini D., Chiuminatto U., Stahl-Zeng J., Orlandini S., Furlanetto S., Del Bubba M. (2017). Liquid chromatographic/electrospray ionization quadrupole/time of flight tandem mass spectrometric study of polyphenolic composition of different Vaccinium berry species and their comparative evaluation. Anal. Bioanal. Chem..

[2] Kula M., Majdan M., Głód D., Krauze-Baranowska M. (2016). Phenolic composition of fruits from different cultivars of red and black raspberries grown in Poland. J. Food Compos. Anal..

[3] La Barbera G., Capriotti A.L., Cavaliere C., Piovesana S., Samperi R., Chiozzi R.Z., Laganà A. (2017). Comprehensive polyphenol profiling of a strawberry extract (Fragaria× ananassa) by ultra-high-performance liquid chromatography coupled with high-resolution mass spectrometry. Anal. Bioanal. Chem..

[4] Burton-Freeman B.M., Sandhu A.K., Edirisinghe I. (2016). Red raspberries and their bioactive polyphenols: Cardiometabolic and neuronal health links. Adv. Nutr..

[5] Mazzoni L., Perez-Lopez P., Giampieri F., Alvarez-Suarez J.M., Gasparrini M., Forbes-Hernandez T.Y., Quiles J.L., Mezzetti B., Battino M. (2016). The genetic aspects of berries: From field to health. J. Sci. Food Agric..

[6] Del Bubba M., Di Serio C., Renai L., Scordo C.V.A., Checchini L., Ungar A., Tarantini F., Bartoletti R. (2020). Vaccinium myrtillus L. extract and its native polyphenol-recombined mixture have anti-proliferative and pro-apoptotic effects on human prostate cancer cell lines. Phytother. Res..

[7] Domazetovic V., Marcucci G., Falsetti I., Bilia A.R., Vincenzini M.T., Brandi M.L., Iantomasi T. (2020). Blueberry Juice Antioxidants Protect Osteogenic Activity against Oxidative Stress and Improve Long-Term Activation of the Mineralization Process in Human Osteoblast-Like SaOS-2 Cells: Involvement of SIRT1. Antioxidants.

[8] God J., Tate P.L., Larcom L.L. (2010). Red raspberries have antioxidant effects that play a minor role in the killing of stomach and colon cancer cells. Nutr. Res..

[9] Rodriguez-Mateos A., Pino-García R.D., George T.W., Vidal-Diez A., Heiss C., Spencer J.P. (2014). Impact of processing on the bioavailability and vascular effects of blueberry (poly) phenols. Mol. Nutr. Food Res..

[10] Park E., Edirisinghe I., Wei H., Vijayakumar L.P., Banaszewski K., Cappozzo J.C., Burton-Freeman B. (2016). A dose–response evaluation of freeze-dried strawberries independent of fiber content on metabolic indices in abdominally obese individuals with insulin resistance in a randomized, single-blinded, diet-controlled crossover trial. Mol. Nutr. Food Res..

[11] Lee J., Dossett M., Finn C.E. (2012). Rubus fruit phenolic research: The good, the bad, and the confusing. Food Chem..

[12] Hidalgo G.-I., Almajano M.P. (2017). Red fruits: Extraction of antioxidants, phenolic content, and radical scavenging determination: A review. Antioxidants.

[13] Pinczinger D., Reth M.v., Keilwagen J., Berner T., Peil A., Flachowsky H., Emeriewen O.F. (2020). Mapping of the Waxy Bloom Gene in ‘Black Jewel’in a Parental Linkage Map of ‘Black Jewel’×‘Glen Ample’(Rubus) Interspecific Population. Agronomy.

[14] FAOSTAT Food and Agriculture Organization of United Nations. http://www.fao.org/faostat/en/#data/QC.

[15] Teegarden M.D., Schwartz S.J., Cooperstone J.L. (2019). Profiling the impact of thermal processing on black raspberry phytochemicals using untargeted metabolomics. Food Chem..

[16] Paudel L., Wyzgoski F.J., Giusti M.M., Johnson J.L., Rinaldi P.L., Scheerens J.C., Chanon A.M., Bomser J.A., Miller A.R., Hardy J.K. (2014). NMR-based metabolomic investigation of bioactivity of chemical constituents in black raspberry (*Rubus occidentalis* L.) fruit extracts. J. Agric. Food Chem..

[17] Mazur S.P., Nes A., Wold A.-B., Remberg S.F., Aaby K. (2014). Quality and chemical composition of ten red raspberry (*Rubus idaeus* L.) genotypes during three harvest seasons. Food Chem..

[18] Dincheva I., Badjakov I., Kondakova V., Dobson P., Mcdougall G., Stewart D. (2013). Identification of the phenolic components in Bulgarian raspberry cultivars by LC-ESI-MSn. Int. J. Agric. Sci. Res..

[19] Mullen W., Lean M.E., Crozier A. (2002). Rapid characterization of anthocyanins in red raspberry fruit by high-performance liquid chromatography coupled to single quadrupole mass spectrometry. J. Chromatogr. A.

[20] Remberg S.F., Sønsteby A., Aaby K., Heide O.M. (2010). Influence of postflowering temperature on fruit size and chemical composition of Glen Ample raspberry (*Rubus idaeus* L.). J. Agric. Food Chem..

[21] Borges G., Degeneve A., Mullen W., Crozier A. (2009). Identification of flavonoid and phenolic antioxidants in black currants, blueberries, raspberries, red currants, and cranberries. J. Agric. Food Chem..

[22] Bradish C.M., Perkins-Veazie P., Fernandez G.E., Xie G., Jia W. (2011). Comparison of flavonoid composition of red raspberries (Rubus idaeus L.) grown in the Southern United States. J. Agric. Food Chem..

[23] Zhang X., Sandhu A., Edirisinghe I., Burton-Freeman B. (2018). An exploratory study of red raspberry (*Rubus idaeus* L.)(poly) phenols/metabolites in human biological samples. Food Funct..

[24] Ancillotti C., Ulaszewska M., Mattivi F., Del Bubba M. (2019). Untargeted Metabolomics Analytical Strategy Based on Liquid Chromatography/Electrospray Ionization Linear Ion Trap Quadrupole/Orbitrap Mass Spectrometry for Discovering New Polyphenol Metabolites in Human Biofluids after Acute Ingestion of Vaccinium myrtillus Berry Supplement. J. Am. Soc. Mass Spectrom..

[25] Carvalho E., Franceschi P., Feller A., Palmieri L., Wehrens R., Martens S. (2013). A targeted metabolomics approach to understand differences in flavonoid biosynthesis in red and yellow raspberries. Plant Physiol. Biochem..

[26] Renai L., Tozzi F., Scordo C.V., Giordani E., Bruzzoniti M.C., Fibbi D., Mandi L., Ouazzani N., Del Bubba M. (2021). Productivity and nutritional and nutraceutical value of strawberry fruits (*Fragaria x ananassa* Duch.) cultivated under irrigation with treated wastewaters. J. Sci. Food Agric..

[27] Del Bubba M., Giordani E., Ancillotti C., Petrucci W.A., Ciofi L., Morelli D., Marinelli C., Checchini L., Furlanetto S. (2016). Morphological, nutraceutical and sensorial properties of cultivated Fragaria vesca L. berries: Influence of genotype, plant age, fertilization treatment on the overall fruit quality. Agric. Food Sci..

[28] Tozzi F., Legua P., Martínez-Nicolás J.J., Núñez-Gómez D., Giordani E., Melgarejo P. (2020). Morphological and nutraceutical characterization of six pomegranate cultivars of global commercial interest. Sci. Hortic..

[29] Douglas C.E., Michael F.A. (1991). On distribution-free multiple comparisons in the one-way analysis of variance. Commun. Stat. Theory Methods.

[30] Sumner L.W., Amberg A., Barrett D., Beale M.H., Beger R., Daykin C.A., Fan T.W.-M., Fiehn O., Goodacre R., Griffin J.L. (2007). Proposed minimum reporting standards for chemical analysis. Metabolomics.

[31] Krüger E., Dietrich H., Schöpplein E., Rasim S., Kürbel P. (2011). Cultivar, storage conditions and ripening effects on physical and chemical qualities of red raspberry fruit. Postharvest Biol. Technol..

[32] Pantelidis G.E., Vasilakakis M., Manganaris G.A., Diamantidis G. (2007). Antioxidant capacity, phenol, anthocyanin and ascorbic acid contents in raspberries, blackberries, red currants, gooseberries and Cornelian cherries. Food Chem..

[33] Stavang J.A., Freitag S., Foito A., Verrall S., Heide O.M., Stewart D., Sønsteby A. (2015). Raspberry fruit quality changes during ripening and storage as assessed by colour, sensory evaluation and chemical analyses. Sci. Hortic..

[34] Wang S.Y., Chen C.-T., Wang C.Y. (2009). The influence of light and maturity on fruit quality and flavonoid content of red raspberries. Food Chem..

[35] Schulz M., Chim J.F. (2019). Nutritional and bioactive value of Rubus berries. Food Biosci..

[36] Del Bubba M., Checchini L., Chiuminatto U., Doumett S., Fibbi D., Giordani E. (2012). Liquid chromatographic/electrospray ionization tandem mass spectrometric study of polyphenolic composition of four cultivars of *Fragaria vesca* L. berries and their comparative evaluation. J. Mass Spectrom..

[37] Hager T.J., Howard L.R., Liyanage R., Lay J.O., Prior R.L. (2008). Ellagitannin composition of blackberry as determined by HPLC-ESI-MS and MALDI-TOF-MS. J. Agric. Food Chem..

[38] Mullen W., Yokota T., Lean M.E., Crozier A. (2003). Analysis of ellagitannins and conjugates of ellagic acid and quercetin in raspberry fruits by LC–MSn. Phytochemistry.

[39] McDougall G., Martinussen I., Stewart D. (2008). Towards fruitful metabolomics: High throughput analyses of polyphenol composition in berries using direct infusion mass spectrometry. J. Chromatogr. B.

[40] Mullen W., McGinn J., Lean M.E., MacLean M.R., Gardner P., Duthie G.G., Yokota T., Crozier A. (2002). Ellagitannins, flavonoids, and other phenolics in red raspberries and their contribution to antioxidant capacity and vasorelaxation properties. J. Agric. Food Chem..

[41] Teixeira N., Azevedo J., Mateus N., de Freitas V. (2016). Proanthocyanidin screening by LC–ESI-MS of Portuguese red wines made with teinturier grapes. Food Chem..

[42] Carvalho E., Fraser P.D., Martens S. (2013). Carotenoids and tocopherols in yellow and red raspberries. Food Chem..

[43] Justino G.C., Borges C.M., Florêncio M.H. (2009). Electrospray ionization tandem mass spectrometry fragmentation of protonated flavone and flavonol aglycones: A re-examination. Rapid Commun. Mass Spectrom..

[44] Yuan T., Guo X.-F., Shao S.-Y., An R.-M., Wang J., Sun J. (2020). Characterization and identification of flavonoids from Bambusa chungii leaves extract by UPLC-ESI-Q-TOF-MS/MS. Acta Chromatogr..

[45] Mikulic-Petkovsek M., Slatnar A., Stampar F., Veberic R. (2012). HPLC–MSn identification and quantification of flavonol glycosides in 28 wild and cultivated berry species. Food Chem..

[46] Bobinaitė R., Viškelis P., Venskutonis P.R. (2012). Variation of total phenolics, anthocyanins, ellagic acid and radical scavenging capacity in various raspberry (*Rubus* spp.) cultivars. Food Chem..

[47] Kaume L., Howard L.R., Devareddy L. (2011). The blackberry fruit: A review on its composition and chemistry, metabolism and bioavailability, and health benefits. J. Agric. Food Chem..

[48] Regos I., Urbanella A., Treutter D. (2009). Identification and quantification of phenolic compounds from the forage legume sainfoin (Onobrychis viciifolia). J. Agric. Food Chem..

[49] Chen L., Xin X., Yuan Q., Su D., Liu W. (2014). Phytochemical properties and antioxidant capacities of various colored berries. J. Sci. Food Agric..

[50] Fu Y., Zhou X., Chen S., Sun Y., Shen Y., Ye X. (2015). Chemical composition and antioxidant activity of Chinese wild raspberry (*Rubus hirsutus* Thunb.). Food Sci. Technol..

[51] Weber C., Perkins-Veazie P., Moore P., Howard L. (2008). Variability of antioxidant content in raspberry germplasm. Acta Hortic..

[52] Tóth G., Barabás C., Tóth A., Kéry Á., Béni S., Boldizsár I., Varga E., Noszál B. (2016). Characterization of antioxidant phenolics in Syringa vulgaris L. flowers and fruits by HPLC-DAD-ESI-MS. Biomed. Chromatogr..

[53] de Andrade Neves N., Stringheta P.C., Gómez-Alonso S., Hermosín-Gutiérrez I. (2018). Flavonols and ellagic acid derivatives in peels of different species of jabuticaba (*Plinia* spp.) identified by HPLC-DAD-ESI/MSn. Food Chem..

[54] Guo S., Duan J.-A., Tang Y.-P., Yang N.-Y., Qian D.-W., Su S.-L., Shang E.-X. (2010). Characterization of triterpenic acids in fruits of Ziziphus species by HPLC-ELSD-MS. J. Agric. Food Chem..

[55] Wang Y., Suo Y., Sun Y., You J. (2016). Determination of triterpene acids from 37 different varieties of raspberry using pre-column derivatization and HPLC fluorescence detection. Chromatographia.

[56] Chai L., Li Y., Chen S., Perl A., Zhao F., Ma H. (2014). RNA sequencing reveals high resolution expression change of major plant hormone pathway genes after young seedless grape berries treated with gibberellin. Plant Sci..

[57] Pérez F.J., Viani C., Retamales J. (2000). Bioactive gibberellins in seeded and seedless grapes: Identification and changes in content during berry development. Am. J. Enol. Vitic..

[58] Palonen P., Pehkonen E., Rantanen M. (2013). Growth control of “Glen Ample” and “Tulameen” raspberry cultivars with single and repeated ProCa applications. Eur. J. Hortic. Sci..

