# Supplementary material for: Liquid Chromatographic Quadrupole Time-of-Flight Mass Spectrometric Untargeted Profiling of (Poly)phenolic Compounds in Rubus idaeus L. and Rubus occidentalis L. Fruits and Their Comparative Evaluation
Source: Antioxidants (Basel). 2021 Apr 29;10(5):704. doi: 10.3390/antiox10050704 (PMC8145548; doi:10.3390/antiox10050704)
Supplement: Supplementary file 1 [file antioxidants-10-00704-s001.zip › antioxidants-1181813-supplementary.pdf]

SUPPLEMENTARY MATERIAL

Section 1–Reference standards

Polyphenol standards were supplied as follows: 3,4-dihydroxybenzoic acid, vanillic acid, ellagic acid, gallic acid coumaric acid caffeic acid, dihydrocaffeic acid, ferulic acid, sinapic acid, chlorogenic acid, neochlorogenic acid, cryptochlorogenic acid, salicylic acid, naringenin-7-O-glucoside, epicatechin, epicatechin gallate, procyanidin B1, procyanidin B2, procyanidin C1, kaempferol, kaempferol-3-O-rutinoside, kaempferol-3-O-glucoside, taxifolin, quercetin, quercetin-3-O-glucoside, quercetin-3-O-galactoside, quercetin-3-O-glucose-6-acetate, quercetin-3,4-diglucoside, quercetin-3-O-glucoronide, quercetin-3-O-sophoroside, myricetin-3-O-glucoside, luteolin, narirutin, polydatin, quercitrine, dicaffeoylquinic acid, esculetin, scopoletin, procyanidin A2, phloretin-2'-O-glucoside (phloridzin), quercetin-3-O-rutinoside, catechin, myricetin, phloretin, galacturonic acid, epigallocathechin gallate, epigallocathechin, coniferyl alcohol 98%, isorhamnetin and isohramnetin-3-O-glucoside by Sigma–Aldrich (St. Louis, MO, USA); cyanidin-3-O-sophoroside, cyanidin-3-O-glucoside, cyanidin-3-O-rutinoside, delphinidin-3-O-glucoside, delphinidin-3-O-galactoside, cyanidin-3-galactoside, cyanidin-3-O-arabinoside, peonidin-3-O-glucoside by Polyphenols Laboratories AS (Sandnes, Norway).

Section 2–Analysis of total soluble polyphenols and total monomeric anthocyanins

Total soluble polyphenols (TSP) and total monomeric anthocyanins (TMA) were spectrophotometrically determined as following described. TSP: 100-200 μL of the extract (depending on the polyphenol concentration in the extract) were mixed with 200 μL of Folin-Ciocalteau reagent. After 3 min, 400 μL of an aqueous solution saturated with sodium carbonate were added and the mixture obtained was made up to 10 mL with ultrapure water. The solution was dark incubated for 1 h; afterwards the absorbance was measured at 740 nm and polyphenol concentration calculated on the basis of a procyanidin B1 calibration curve. TMA: aliquots of 100-200 μL of berry extract were diluted in buffer solutions at pH=1 and pH=4.5, so as to obtain a final volume of 10 mL. The absorbance (Abs) of both solutions were measured at 520 and 700 nm and the quantity "ΔAbs" was calculated according to equation 1.

$$\Delta Abs=\left(Abs_{pH=1}^{520\ nm}-Abs_{pH=1}^{700\ nm}\right)-\left(Abs_{pH=4.5}^{520\ nm}-Abs_{pH=4.5}^{700\ nm}\right) \quad (1)$$

Similarly, "ΔAbs" values were also calculated for different concentrations of cyanidin-3-O-sophoroside reference standard and plotted as a function of the corresponding concentrations. The best equation fitting the experimental points was calculated by the least square method, thus obtaining a linear calibration curve. TMA in the extracts were finally calculated using this calibration curve.

Section 3–Feature annotation

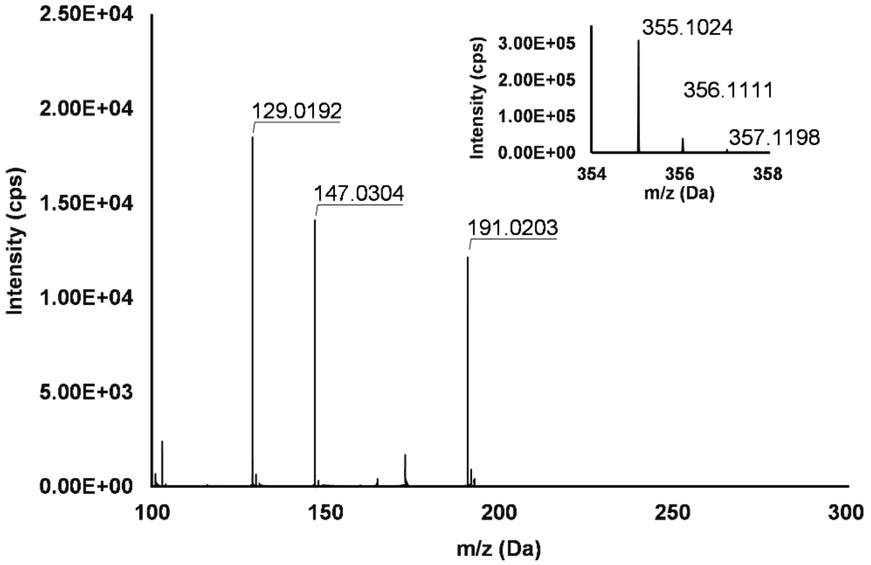

Figure S1–TOF MS (upper right) and Q/TOF MS[2] spectra of peaks 6 and 9 tentatively identified as ferulic acid hexosides.

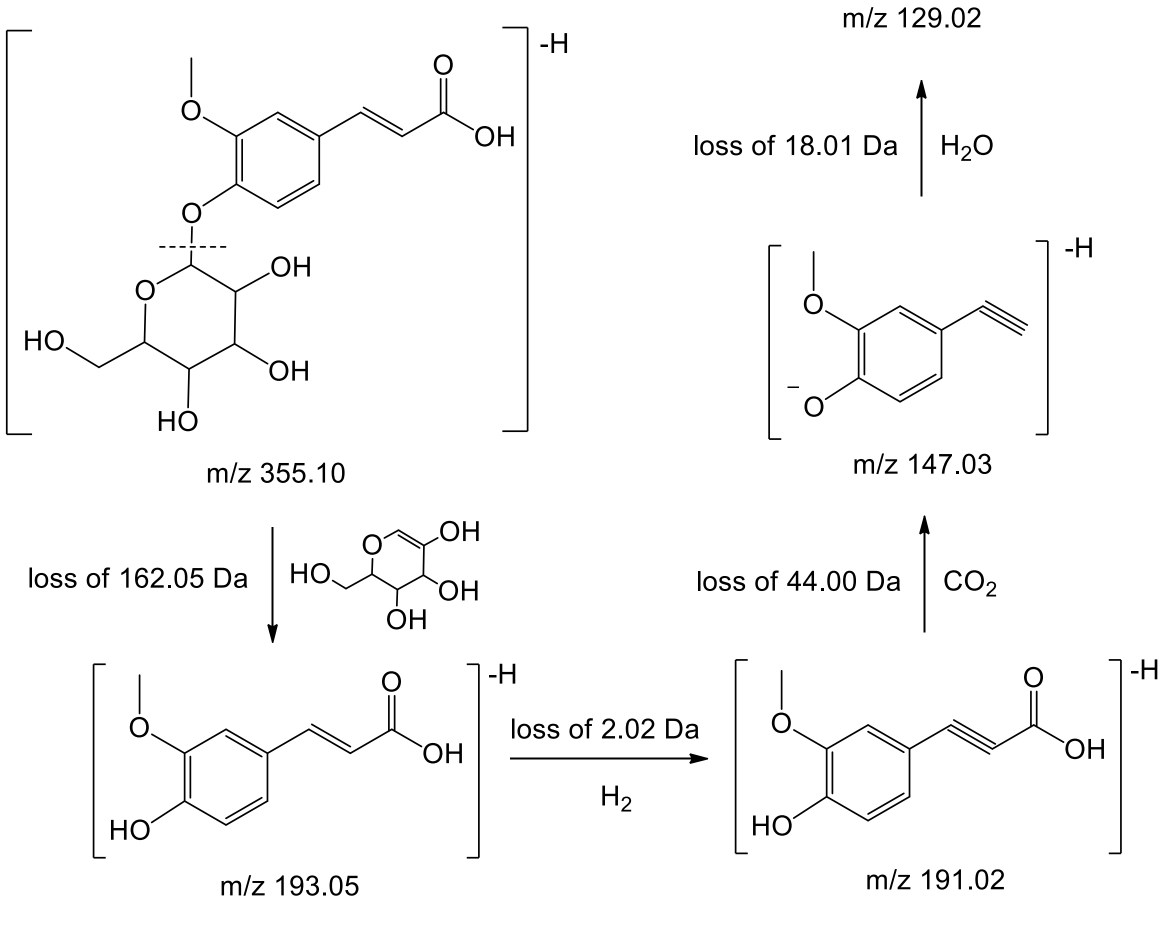

Scheme S1–Hypothesized structure and fragmentation scheme for peaks 6 and 9 ($[M–H]^-$ = 355.1024 and 355.1026).

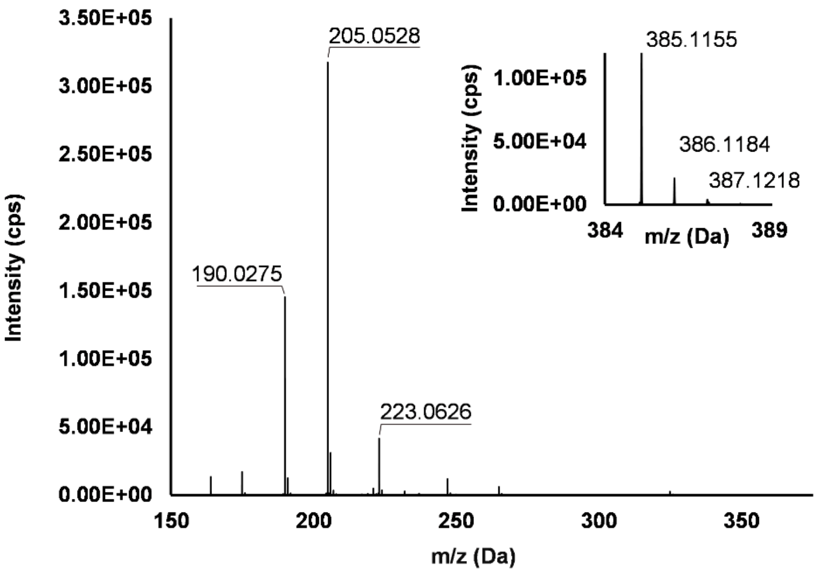

Figure S2–TOF MS (upper right) and Q/TOF MS^2 spectra of peak 27 tentatively identified as sinapic acid hexosides.

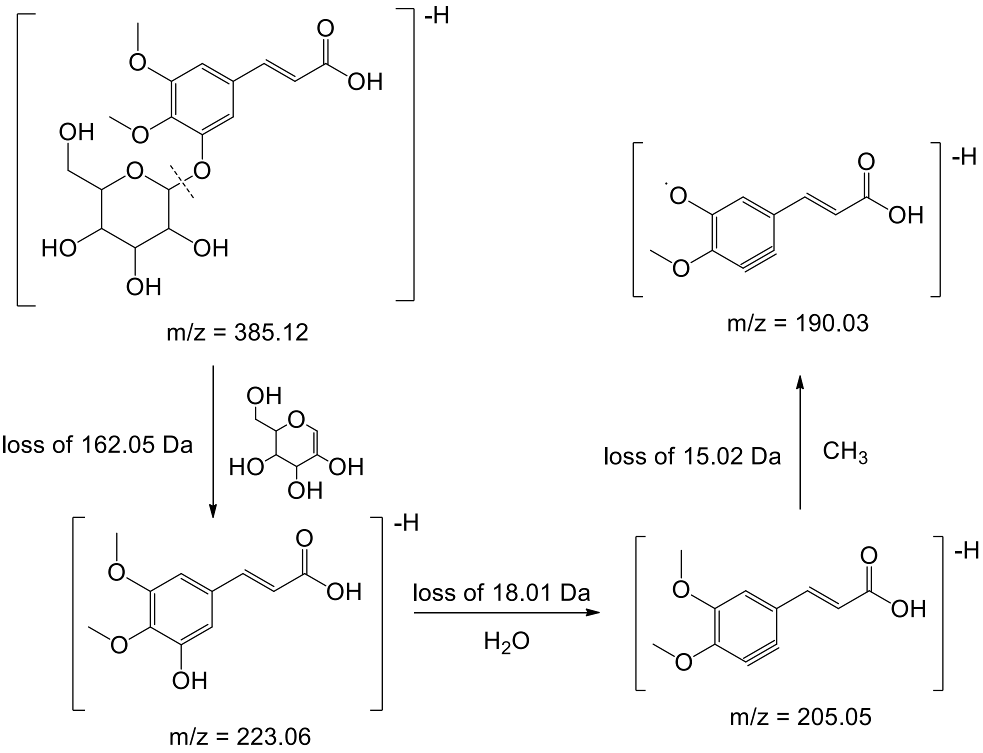

Scheme S2–Hypothesized structure and fragmentation scheme for peak 27 ([M–H]$^-$ = 385.1154).

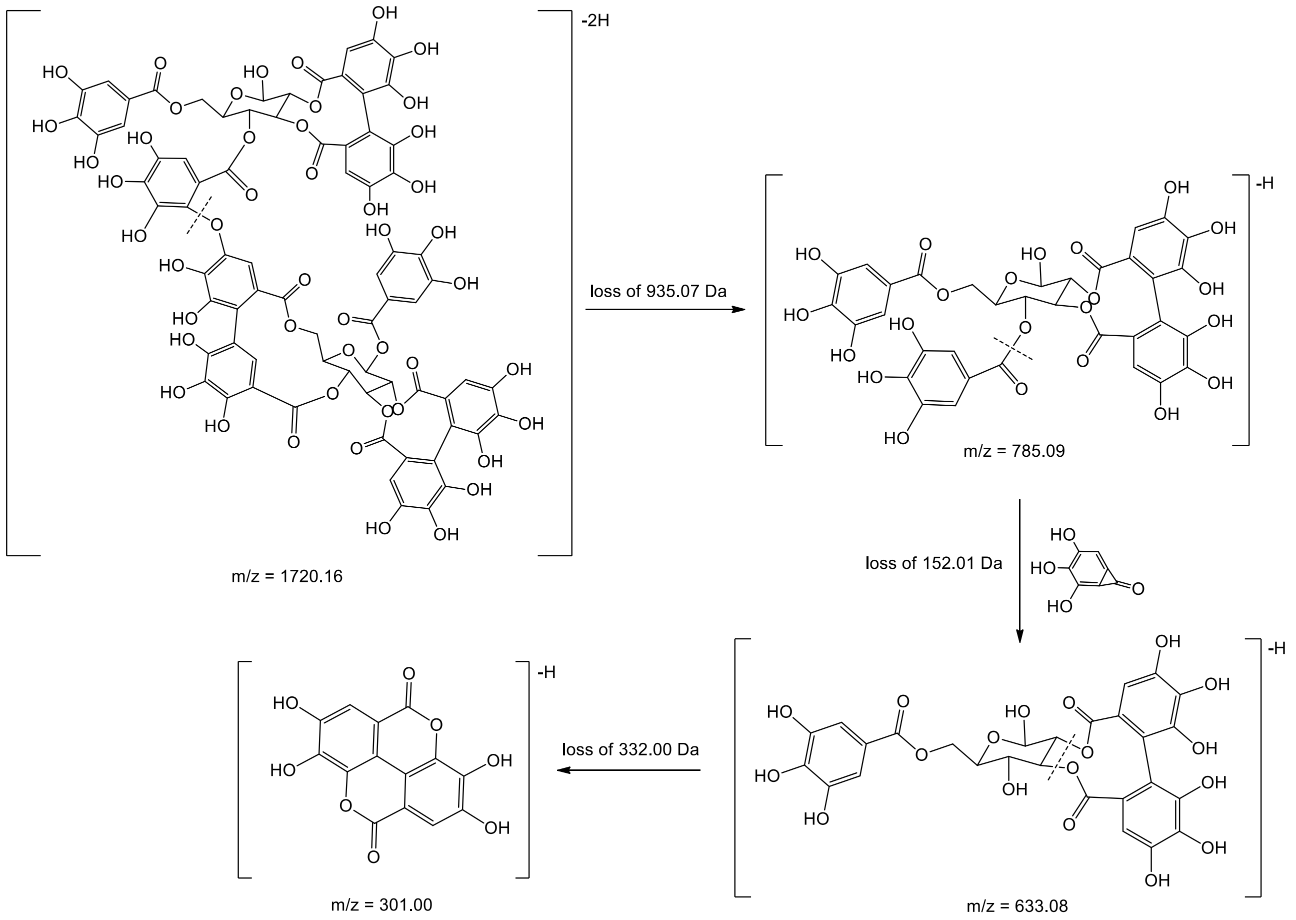

Scheme S3–Hypothesized structure and fragmentation scheme for peak 28 ($[M–2H]^{2–}/2 = 859.0802$).

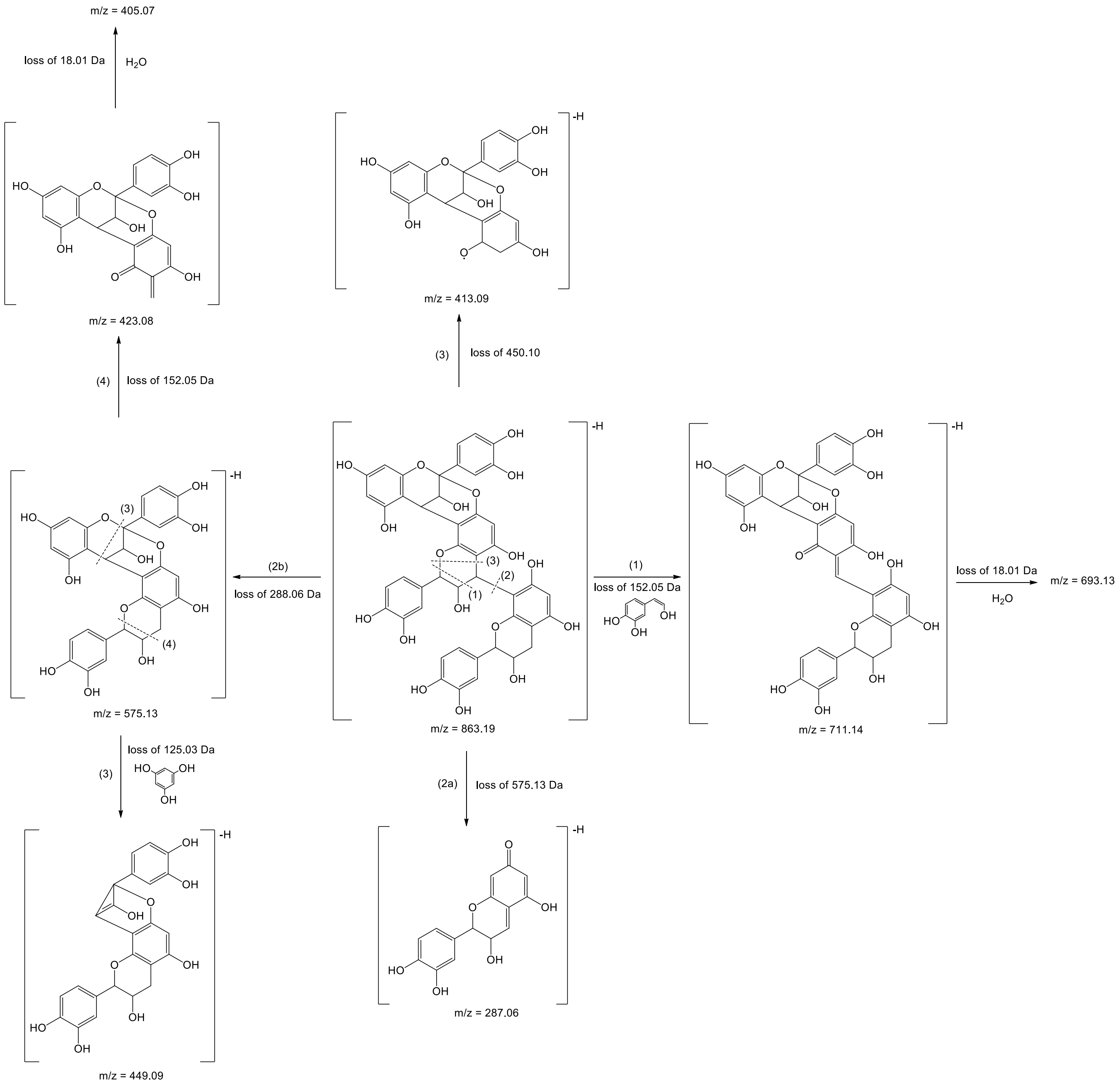

Scheme S4–Hypothesized structure and fragmentation scheme for peak 11 ([M–H]$^{-}$ = 863.1903).

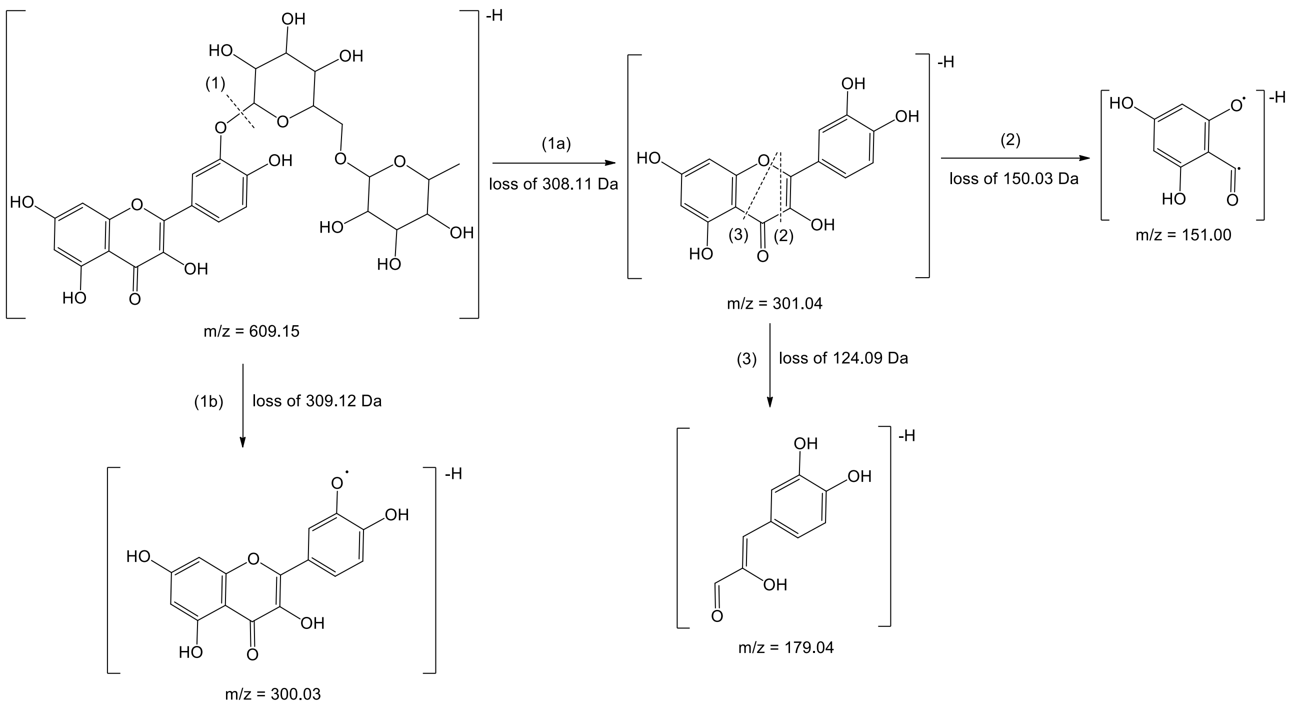

Scheme S5–Hypothesized structure and fragmentation scheme for peak 13 ([M–H]$^-$ = 609.1499).

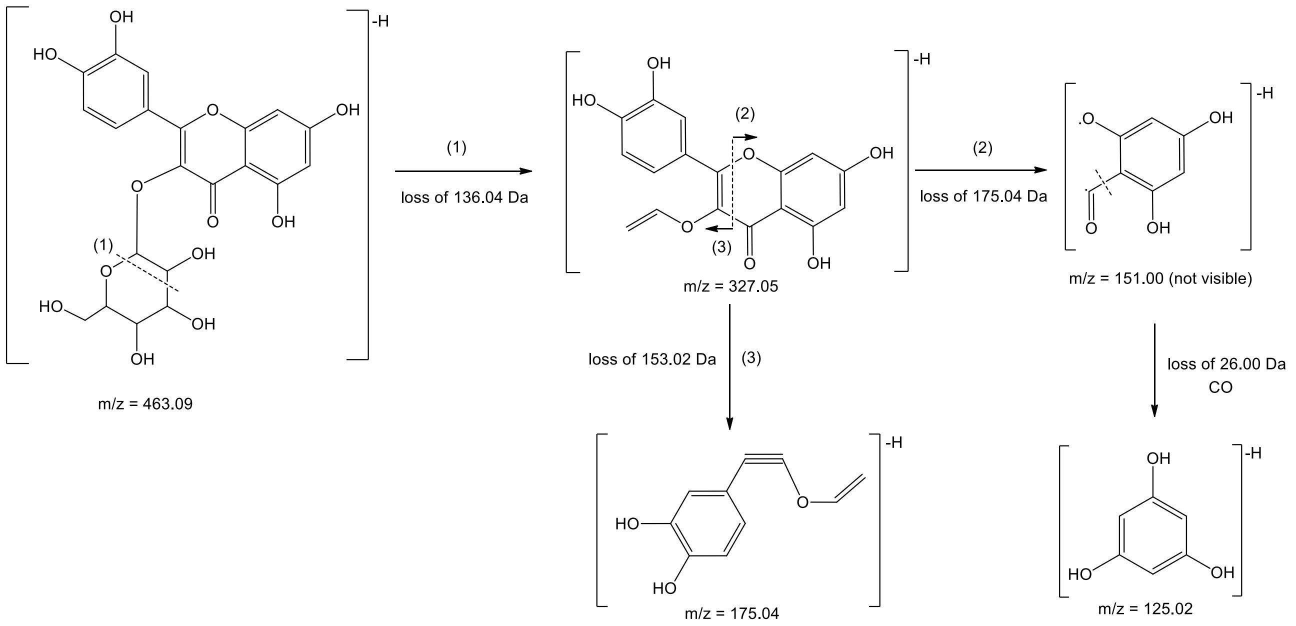

Scheme S6–Hypothesized structure and fragmentation scheme for peak 15 ([M–H]$^-$ = 463.0892).

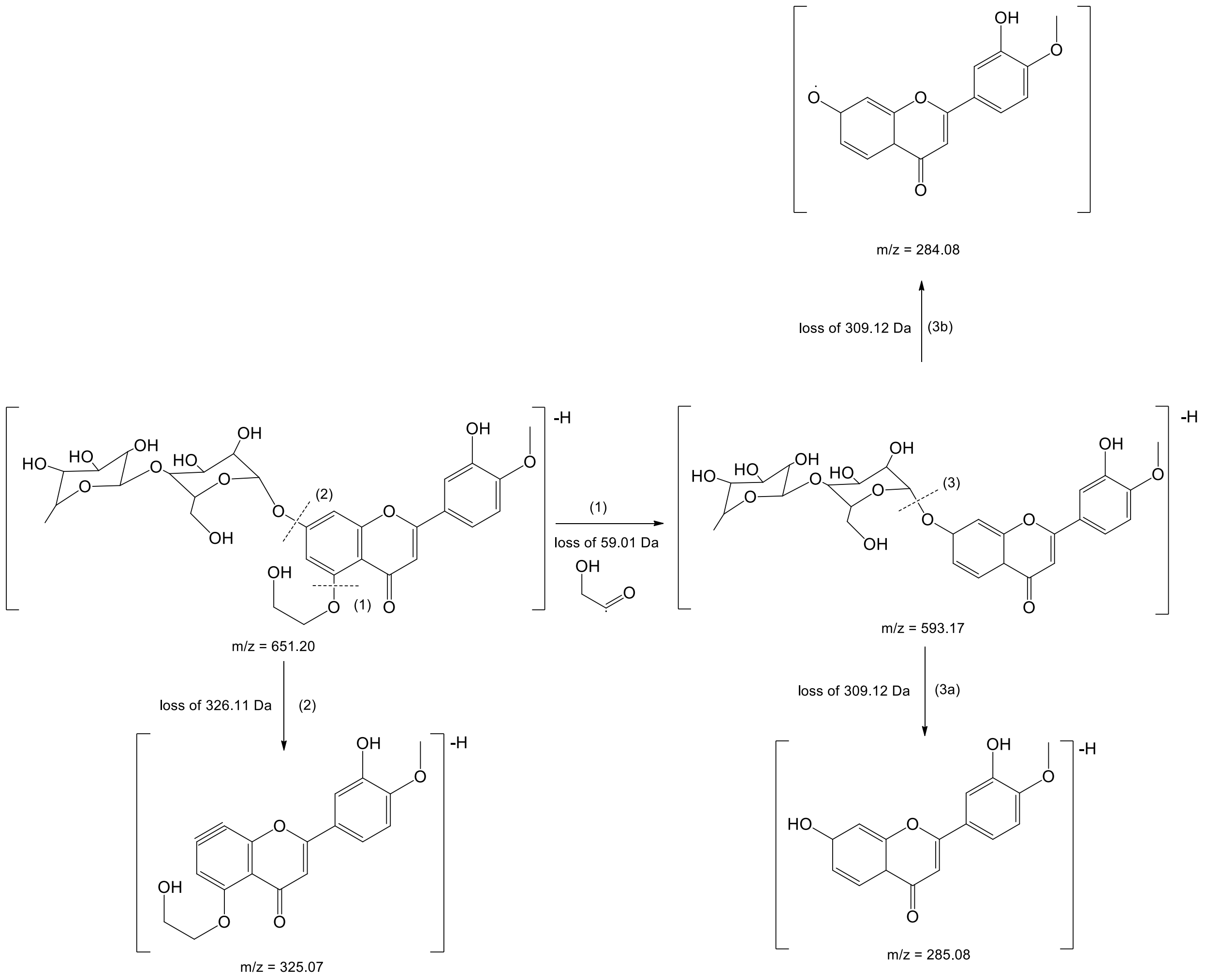

Scheme S7–Hypothesized structure and fragmentation scheme for peak 30 ([M–H]$^{-}$ = 651.1963).

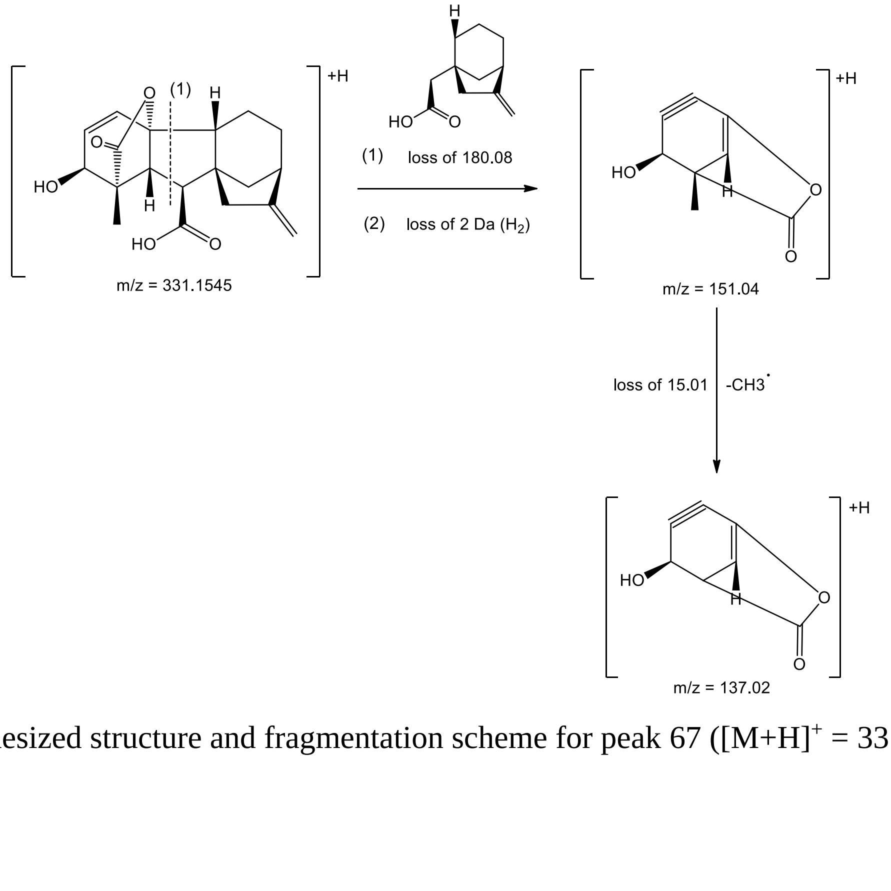

Scheme S8–Hypothesized structure and fragmentation scheme for peak 67 ([M+H]$^{+}$ = 331.1545).
